# An unattended image-processing pipeline for on-the-fly quality assessment and 3D exploration in cryo-EM

**DOI:** 10.1107/S205979832600656X

**Published:** 2026-07-28

**Authors:** Daniel Marchán Torres, Pablo Conesa, Alberto Garcia, Mikel Iceta, Ludovic Broche, Marcos Gragera, Romain Linares, Hok Sau Kwong, Francisco Javier Chichón, Olof Svensson, Rocío Arranz, Grégory Effantin, Eaazhisai Kandiah, José María Carazo, Carlos O. S. Sorzano

**Affiliations:** a Centro Nacional de Biotecnologia, Consejo Superior de Investigaciones Cientificas (CSIC), Spain; b The European Synchrotron (ESRF), France; c European Molecular Biology Laboratory (EMBL) Grenoble, France; d Université Grenoble Alpes, CNRS, CEA, Institut de Biologie Structurale (IBS), France; Duke University, USA

**Keywords:** cryo-EM, SPA, on-the-fly processing, automation, *Scipion*

## Abstract

Single-particle analysis (SPA) by cryogenic electron microscopy (cryo-EM) has become a cornerstone of structural biology; yet, the workflow from data acquisition to a 3D structure remains a time-consuming, manual and significant task. Researchers often invest days of valuable microscope time collecting massive datasets with little to no real-time feedback on sample quality or the ultimate feasibility of achieving a high-resolution reconstruction. To address this challenge, we have developed an automated, end-to-end processing workflow designed for on-the-fly analysis. Built within the *Scipion* framework, the pipeline integrates a cascade of automated quality-control filters, a novel consensus-based strategy for training a data-specific particle-picking model, and a parallel 2D/3D validation scheme to ensure robust processing outcomes. We validated the workflow on a diverse benchmark of 32 datasets (CryoPPP), achieving a 94% overall processing success rate and obtaining high-quality 3D reconstructions in 78% of cases. Real-world deployment at the European Synchrotron (ESRF) cryo-EM facility further validated its practical utility: the pipeline demonstrated processing speeds exceeding data acquisition, delivering preliminary 3D maps in under 3 h. Approximately 70% of user experiments converged to interpretable structures, with half achieving 3–4 Å resolution, despite the inherent complexity of facility-collected samples. By providing researchers with rapid, actionable feedback and identifying both promising and problematic datasets in real time, this workflow transforms cryo-EM data collection from a passive process into an active, data-driven experiment. It serves as a powerful diagnostic and decision-support tool, accelerating structural determination and optimizing microscope time in high-throughput cryo-EM environments.

## Abstract 2

We present a fully automated, unattended cryo-EM pipeline that serves as an on-the-fly diagnostic tool, enabling rapid data-quality assessment and informed decision-making to maximize data-collection efficiency.

## 1. Introduction

Single-particle analysis (SPA) by cryogenic electron microscopy (cryo-EM) has become a cornerstone of modern structural biology, enabling the determination of near-atomic resolution structures of macromolecules without the need for crystallization (Cheng, 2018 ▸; Dubochet *et al.*, 2017 ▸). The development of direct electron detectors and sophisticated image-processing algorithms has fueled a ‘resolution revolution’, positioning cryo-EM as a dominant technique for studying large, flexible and heterogeneous complexes that were previously intractable (Kühlbrandt, 2014 ▸; Raimondi & Grinzato, 2022 ▸). This technological leap, combined with the automation of data acquisition, has transformed high-end electron microscopes into powerful, high-throughput instruments capable of generating data at rates of several hundred movies per hour, amounting to terabytes in a single session (Baldwin *et al.*, 2018 ▸).

However, this success has shifted the primary bottleneck from data collection to data processing and, more critically, to timely data-quality assessment. Researchers frequently invest one or two days of valuable, scarce microscope time to collect massive datasets with little to no effective real-time feedback on the ultimate feasibility of achieving a high-resolution reconstruction (Mendez *et al.*, 2023 ▸). Waiting until after a session concludes to analyze the data is inefficient and risky, as entire datasets may be collected from suboptimal samples, only to be discarded days later. This has established on-the-fly processing, which provides real-time feedback during acquisition, as an essential tool for maximizing the efficiency and scientific output of modern cryo-EM facilities (Mendez *et al.*, 2023 ▸; Li, Cash *et al.*, 2020 ▸).

To meet this need, several on-the-fly software pipelines have been developed. These can be broadly categorized into flexible, wrapper-based frameworks such as *Scipion* (de la Rosa-Trevín *et al.*, 2016 ▸; Gómez-Blanco *et al.*, 2018 ▸; Conesa *et al.*, 2023 ▸), and more self-contained, all-in-one suites such as *RELION* (Kimanius *et al.*, 2021 ▸) and *CryoSPARC* (Punjani *et al.*, 2017 ▸). While specialized tools such as *Warp* (Tegunov & Cramer, 2019 ▸) focus on pre-processing speed, only a few, such as *TranSPHIRE* (Stabrin *et al.*, 2020 ▸), have attempted a fully automated end-to-end pipeline. These platforms have successfully automated many repetitive tasks, enabling the rapid feedback critical to efficient data collection.

Despite these advances, a closer examination of the state of the art reveals persistent gaps in essential aspects. A primary issue is the expertise bottleneck. Nearly all current pipelines require significant *a priori* knowledge and user manual intervention to function optimally. Critical parameters, such as particle size, which picker to use and various quality-control thresholds, must be set correctly before processing to avoid suboptimal results (Kimanius *et al.*, 2021 ▸; Stabrin *et al.*, 2020 ▸; Maruthi *et al.*, 2020 ▸). Secondly, the field faces a trade-off between flexibility and simplicity. While all-in-one suites are often rigid and simple, building a truly automated workflow within a flexible, extensible framework such as *Scipion*, which allows the integration of best-in-class software, remains a significant and unsolved challenge. Furthermore, most systems lack a cascade of multi-stage quality filters that automatically curate data at every key processing step. Finally, the output of most on-the-fly pipelines is not immediately ready for final analysis; the hand-off to high-resolution refinement is often not seamless and may require reprocessing, failing to provide a truly efficient head start.

Therefore, a critical need remains for a processing pipeline that is not only automated but also intelligent and robust enough to handle a wide variety of samples. Such a system should infer key parameters directly from the data, be built within a flexible framework to ensure that it remains state of the art, and produce a curated dataset that provides a seamless start for final refinement.

Here, we present a fully automated image-processing pipeline within the *Scipion* framework, designed for on-the-fly quality assessment and 3D exploration. Our system serves as a robust diagnostic tool, providing researchers with rapid, actionable insights into their data quality and the feasibility of their experiments. By automating key processing steps through a multi-stage quality-control cascade and by validating its own results, our pipeline addresses the limitations of current approaches. It represents a crucial step towards more intelligent, efficient and accessible structural biology workflows.

## 2. Methods

This section describes a novel, fully automated pipeline for on-the-fly cryo-EM data processing that transforms raw movies into preliminary 3D reconstructions without user intervention, making it particularly suitable for high-throughput facility environments. All algorithms are integrated within the open-source *Scipion* framework, providing ease of use (no coding required), broad accessibility and seamless community integration.

### 2.1. *Scipion*-based processing pipeline overview

*Scipion* is a Python-based workflow engine that integrates a wide range of structural biology software into a cohesive, interoperable environment. Its plugin-based architecture wraps external packages (*e.g.CryoSPARC*, *RELION*, *MotionCor*3, *SPHIRE*, *Xmipp*, *cisTEM*) into standardized plugins that expose individual image-processing steps as modular protocols. Each protocol manages its own inputs, outputs and parameter conversions, enabling protocols from different software packages to be seamlessly connected into coherent processing workflows. Workflows are defined as JSON templates that encode the full processing graph, including parameter settings and data dependencies. Templates can be imported into new *Scipion* instances to rapidly instantiate new projects or replicate identical pipelines across datasets and across different *Scipion* installations, facilitating reproducibility and standardized processing.

**Figure 1:**
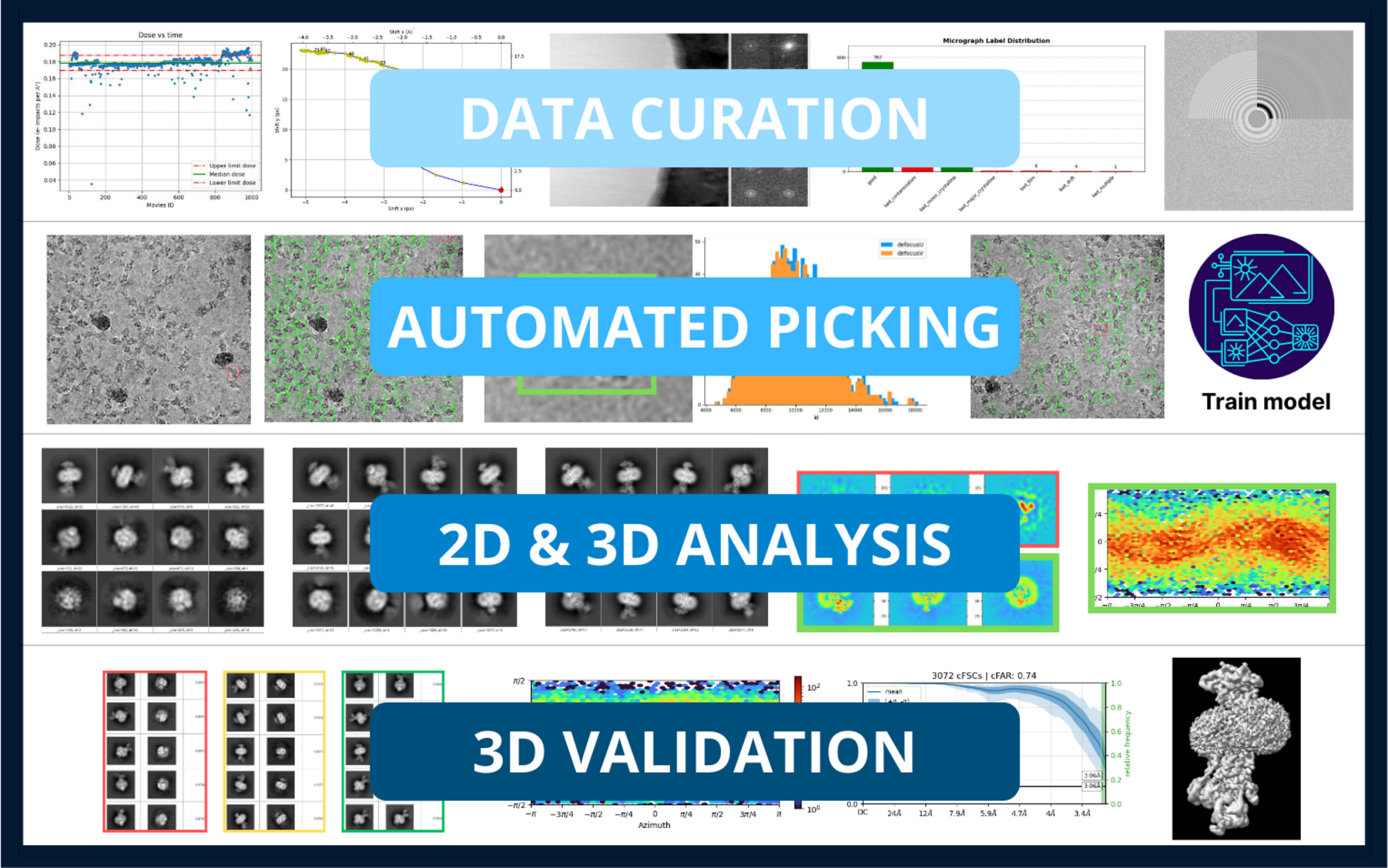
Graphical summary of the four main stages of the automated image-processing pipeline.

Orchestrated within this environment, the proposed pipeline integrates 37 protocols from 11 software packages, all accessed through *Scipion* plugins, and supports execution in streaming mode, allowing processing to begin during data acquisition. A comprehensive list of all software versions, their functional roles and the specific pre-trained model weights used for deep learning-based algorithms is provided in Section S1. Computational tasks can be scheduled using job schedulers such as *SLURM*, ensuring scalability from workstations to high-performance computing (HPC) clusters. The pipeline is organized into four principal stages (Fig. 1 ▸):

(i) *Data curation and quality control*: initial assessment and filtering of raw data.
(ii) *Automated particle picking and model training*: identification and extraction of particles from curated micrographs.
(iii) *Initial 2D and 3D analysis*: 2D classification of particles and parallel generation of *de novo* 3D models.
(iv) *Refinement and parallel validation*: refinement of initial 3D models, coupled with robust validation procedures.

A more detailed representation of the pipeline is provided in Supplementary Fig. S1.

### 2.2. Data curation and quality control

**Figure 2:**
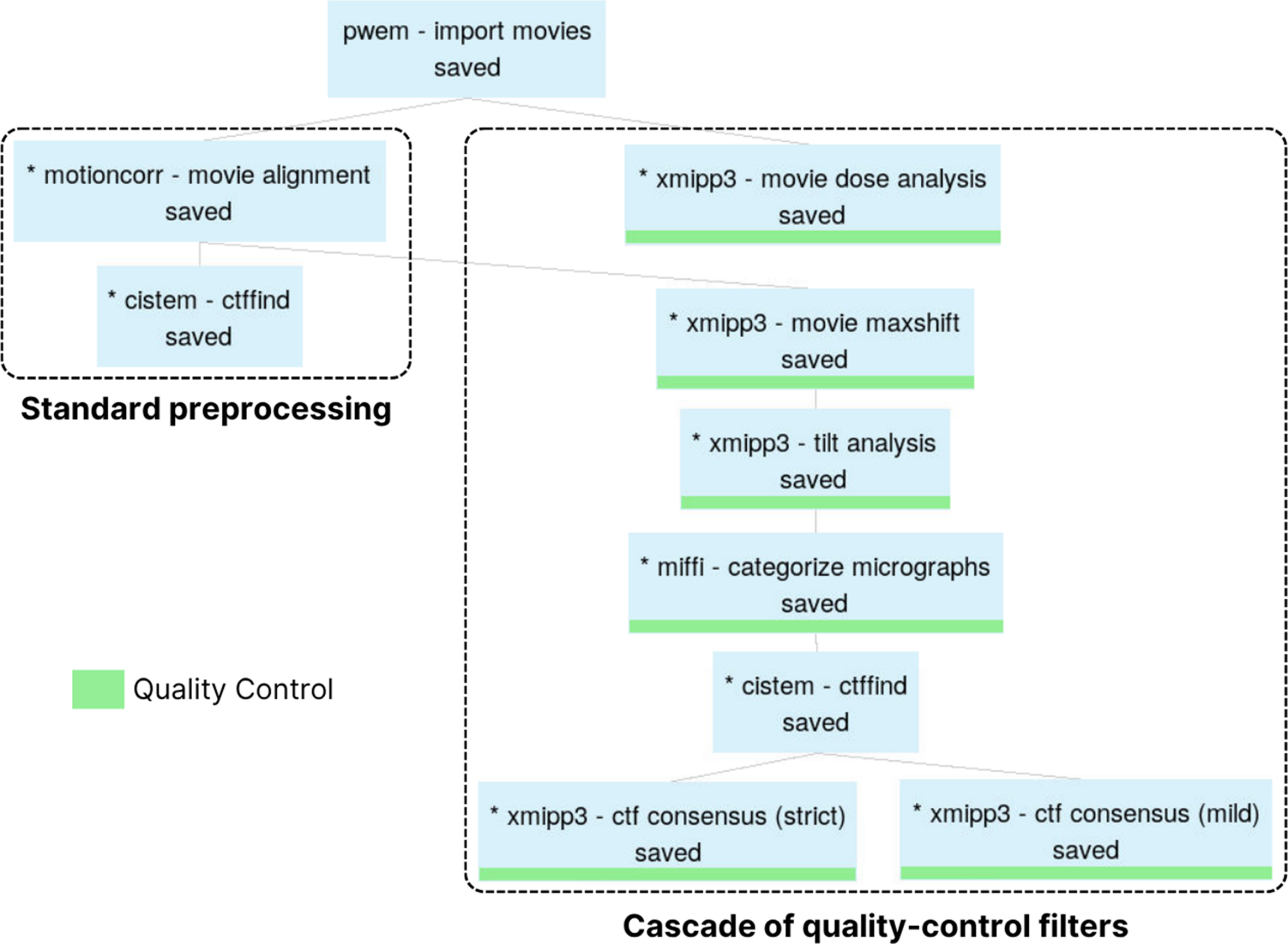
Detailed *Scipion* workflow diagram for the data-curation stage. Each box represents a *Scipion* protocol corresponding to a specific image-processing step in the pipeline. The diagram is divided into two main sections: standard preprocessing, including motion correction and CTF estimation, and a cascade of quality-control filters. The quality filters used during data curation are highlighted in green. The strict CTF consensus is used to estimate particle diameter, while the mild CTF consensus serves as the final filter for selecting curated micrographs. The strict and mild CTF consensus criteria correspond to different parameter thresholds applied during filtering.

The first stage of the pipeline is critical for the success of any high-resolution reconstruction. It comprises a cascade of automated filtering steps designed to ensure that only data likely to contribute meaningful structural information proceed to downstream analysis (Fig. 2 ▸). This is achieved by assessing raw movies and aligned micrographs using information from both real and Fourier space.

#### 2.2.1. Movie-level analysis

Before frame alignment, the raw movies contain valuable information about acquisition stability and ice thickness. Assessing these parameters at the movie level enables the early identification of problematic data.

##### 2.2.1.1. Movie dose analysis protocol

An ideal data acquisition involves a stable and consistent electron dose delivered to the sample throughout the exposure time. Significant fluctuations in dose can indicate instability in the microscope’s illumination system (*i.e.* the electron gun), enabling early intervention and preventing data collection under suboptimal conditions. In addition, dose measurements serve as a valuable proxy for the thickness of the vitrified ice layer: thicker ice scatters more electrons, resulting in a lower average number of electrons reaching the detector for a given incident beam intensity. By measuring the mean dose per frame for each movie and plotting the values over the course of a session, trends and outliers can quickly be identified, allowing the detection of grid areas with inconsistent ice thickness, a common factor that can compromise the final resolution of the 3D reconstruction.

We developed a program to monitor dose stability and infer real-time variations in ice thickness. The program calculates the mean dose per square ångström (e^−^ Å^−2^) for each movie by sampling only the first, middle and last frames, thereby optimizing performance. The mean pixel intensity of these frames is converted to a dose value and averaged to yield a representative dose, *d*_*i*_, for each movie *M*_*i*_.

An initial batch of movies (*n*_samples_, typically 20) is used to establish a robust global reference dose μ, calculated as the median of their representative doses. A subsequent movie *M*_*k*_ with mean dose *d*_*k*_ is accepted if it falls within a user-defined percentage threshold, τ, of the reference:
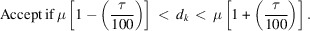


To adapt to gradual changes in experimental conditions, the reference dose μ is updated periodically using the median of all representative doses observed so far. The use of the median ensures resilience to outliers and sporadic deviations. A sliding window, defined as a fixed-size buffer containing the last *N* processed movies, is used to compute recent rejection statistics and assess dose consistency. The window size *N* is user-configurable (default *N* = 50). Smaller windows increase sensitivity to abrupt dose changes but may be more sensitive to noise, while larger windows improve robustness to short-term variability but may delay the detection of sudden shifts. For typical sessions (1000–15 000 movies), values of *N* between 50 and 100 provide a balance between responsiveness and stability.

If the rejection rate within the most recent window exceeds a user-defined threshold (default 30%), a warning is logged to alert the operator of potential issues. A visual example comparing optimal acquisition (with a stable and consistent electron dose) with an unstable acquisition is provided in Supplementary Fig. S2. Since optimal ice thickness is protein-dependent, this tool monitors data-collection quality rather than discarding movies outright. The program is integrated into the workflow via the *Xmipp* software plugin (Střelák *et al.*, 2021 ▸), specifically as the movie dose analysis protocol.

#### 2.2.2. Motion correction and drift monitoring

After acquisition, each movie undergoes motion correction to compensate for specimen movement during electron exposure. Accurate correction is crucial, as excessive drift can result in the irreversible loss of high-resolution information, degrading the quality of the final 3D reconstruction (Střelák *et al.*, 2023 ▸). Persistent or severe drift may also indicate mechanical instability in the microscope. Therefore, monitoring drift magnitude and discarding movies with excessive motion is essential for data-quality control.

##### 2.2.2.1. Movie max shift protocol

To quantify drift, we implemented a calculation that runs immediately after frame alignment (*MotionCor*3; Zheng *et al.*, 2017 ▸). Two key metrics are computed from the absolute shifts (*x*_*j*_, *y*_*j*_) of each frame *j* relative to a reference.

(i) *Maximum frame-to-frame shift* (*S*_frame_). This metric identifies sudden movements by quantifying the largest displacement between any two consecutive frames. Let *l*_*j*_ = [(*x*_*j*_ − *x*_*j*−1_)^2^ + (*y*_*j*_ − *y*_*j*−1_)^2^]^1/2^ represent the Euclidean distance (scalar shift) between consecutive frames *j* and *j* − 1. *S*_frame_ is defined as the maximum of these shifts over the entire movie, converted to ångströms using the pixel size *s*: 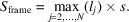
(ii)* Accumulated movie shift* (*S*_movie_). This metric quantifies the total path length traveled by the specimen during exposure, capturing nonlinear and continuous drift more accurately than a simple start-to-end vector. It is calculated as the sum of all incremental shifts *l*_*j*_: 
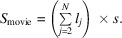

Flexible rejection criteria are provided: movies can be discarded if either threshold is exceeded (OR logic), both thresholds are exceeded (AND logic) or if a single metric is exceeded. For robust data quality, we use OR logic: movies are rejected if either *S*_frame_ or *S*_movie_ exceeds its respective limit.

Both the rejection logic and thresholds are user-configurable, allowing adaptation to different experimental conditions. Default thresholds were empirically determined: for standard data collection, *S*_frame_ = 10 Å and *S*_movie_ = 45 Å. For tilted data, where higher drift is common, relaxed values are used: *S*_frame_ = 30 Å and *S*_movie_ = 120 Å. These thresholds are designed to eliminate outliers with gross drift that preclude the recovery of high-resolution information, rather than borderline cases, and small adjustments do not significantly affect the overall data quality. An example of a micrograph discarded by the movie max shift filter is provided in Supplementary Fig. S3. This program is integrated into the *Xmipp* software plugin as the movie max shift protocol.

#### 2.2.3. Micrograph-level curation

After alignment, micrograph curation is performed using a hybrid approach that leverages information from both Fourier space (via power spectral density, PSD) and real space (via a deep-learning classifier). Analyzing both perspectives provides a more comprehensive assessment of quality than either view alone (Frank, 2006 ▸).

(i) *Real space*. Visual inspection of the micrograph reveals the spatial distribution of particles and the presence of gross contaminants, such as large ice crystals or carbon film edges. It provides the most direct and intuitive assessment.
(ii) *Fourier space (PSD)*. The PSD, defined as the squared magnitude of the micrograph’s Fourier transform, visualizes the image’s spatial frequency components. This view is exceptionally sensitive to periodic signals and global image properties. The most critical feature in the PSD of a high-quality micrograph is the presence of Thon rings: concentric rings corresponding to the zeros of the microscope’s contrast transfer function (CTF). The visibility and radial extent of these rings are direct indicators of a micrograph’s potential resolution: the farther the rings extend from the center, the higher the resolution of the information they contain. Furthermore, the shape and integrity of the Thon rings serve as powerful diagnostic tools. Elliptical rings reveal astigmatism, while directional streaking or blurring in the PSD can indicate uncorrected sample drift. A complete absence of Thon rings implies a severe lack of high-resolution signal, often due to empty foil holes, excessive ice thickness, poor contrast or significant drift (Rohou & Grigorieff, 2015 ▸).

##### 2.2.3.1. Micrograph power spectral density (PSD) analysis protocol

To quantify Fourier-space signal quality, we developed PSD-based consistency metrics that evaluate the spatial uniformity and isotropy of the Thon-ring pattern across each micrograph. The algorithm partitions the micrograph into four corner quadrants. For each quadrant, the following pre­processing steps are applied: (i) normalization to zero mean and unit variance, (ii) computation of the PSD, (iii) cropping to a user-defined resolution limit (default 4 Å) to focus on the most informative frequency range and (iv) low-pass filtering to suppress high-frequency noise and enhance Thon-ring visibility. Two complementary correlation analyses are then performed.

(i) *Cross-quadrant correlation*. The cross-correlation coefficient is computed for all six unique pairs of quadrant PSDs. This metric quantifies the consistency of the Thon-ring pattern across the entire micrograph. High average correlation values indicate uniform imaging conditions (*e.g.* flat ice, consistent defocus, stable illumination), whereas low correlation reflects spatial non-uniformity caused by ice-thickness gradients, partial beam obstruction, specimen tilt or local defocus variations. Cross-correlation provides a robust similarity metric because it is invariant to global intensity scaling and emphasizes structural agreement in frequency space.
(ii) *Rotational autocorrelation*. Each quadrant PSD is correlated with its 90°-rotated version. Circular Thon rings yield high rotational correlation, whereas elliptical rings caused by astigmatism reduce correlation. This metric, therefore, provides a rotation-invariant measure of astigmatism severity and directional distortions in the PSD.

The mean and standard deviation of the resulting ten correlation values (six cross-quadrant and four rotational correlations) are used as final quality scores. A low mean correlation indicates a globally degraded or inconsistent signal (*e.g.* thick ice, empty holes, severe astigmatism or strong drift), whereas a high standard deviation reflects pronounced spatial disparity within the micrograph. Examples of PSD analysis plots for accepted and discarded micrographs, showing different mean correlation values and standard deviations, are provided in Supplementary Fig. S4.

Based on empirical benchmarking across multiple datasets and acquisition conditions, default acceptance thresholds were set to a mean correlation >0.35 and a standard deviation <0.15. These values were chosen as conservative quality gates rather than strict cutoffs, rejecting only micrographs with clearly degraded PSD patterns unlikely to contribute high-resolution information. Adjusting any of the thresholds by ±0.1 makes the filter stricter or more permissive. Stricter thresholds increase rejection rates and may reduce particle yield, whereas more permissive thresholds allow lower quality micrographs to propagate downstream. For this reason, all thresholds are user-configurable and intended as robust defaults applicable across diverse samples rather than dataset-specific parameters.

Furthermore, the resolution-limit cutoff determines which spatial frequencies are included in the analysis. Smaller cutoffs (*e.g.* 4 Å) include higher resolution Thon rings located towards the PSD periphery, which are most sensitive to subtle image degradation. In practice, Thon rings beyond 3–4 Å are rarely visible in routine cryo-EM data, making 4 Å a practical default that balances sensitivity and robustness. Larger cutoffs (*e.g.* >5 Å) restrict the analysis to low-resolution rings only, reducing sensitivity to high-frequency signal loss and making the filter less discriminative. However, in datasets where low resolution is expected, the cutoff should be increased to >5 Å. In such cases, higher resolution Thon rings are typically absent, and attempting to correlate frequencies with no signal may lead to correlations dominated by noise. This scenario would artificially reduce the algorithm’s computed mean correlation values and, consequently, lead to a larger number of micrographs being discarded by the quality filter.

This program is integrated into the *Xmipp* software plugin as the micrographs PSD analysis protocol.

##### 2.2.3.2. *Miffi* for micrograph categorization protocol

To leverage deep learning, the *Miffi* software tool (Xu & Ando, 2024 ▸) was integrated into our workflow via a *Scipion* plugin. *Miffi* employs a dual-branch convolutional neural network (CNN) that simultaneously processes a micrograph’s real-space image and its PSD. This hybrid approach enables the network to learn features related to both particle distribution and CTF quality (such as Thon rings, astigmatism and drift). The network classifies micrographs into ‘Good’ or various ‘Bad’ categories (*e.g.* Crystalline ice, Contamination, Drift) with an accuracy of over 90%. Examples of problematic micrographs for each label category are provided in Fig. 2 of the reference article (Xu & Ando, 2024 ▸), where each real-space micrograph is paired with its corresponding power spectrum. The *Miffi* protocol is designed for high-throughput, streaming-capable processing and utilizes available GPUs for acceleration. For downstream analysis, only micrographs labeled as ‘Good’ or ‘Minor crystalline ice’ are retained by this filter.

#### 2.2.4. CTF estimation filter protocol

As a final quality-control step, a traditional filter is applied using parameters derived from CTF estimation performed with the *CTFFIND*5 algorithm (Elferich *et al.*, 2024 ▸). This program estimates defocus and astigmatism by fitting theoretical CTF models to the power spectrum of each micrograph. The following key parameters are used for filtering.

(i) *CTF fit resolution*. The highest spatial frequency at which the experimental power spectrum significantly correlates with the fitted CTF model. This metric serves as a proxy for the presence of reliable high-resolution signals. For example, very high resolution fits may indicate a strong signal originating from a carbon support film, whereas low-resolution fits can indicate crystalline ice, motion-correction failure or severe radiation damage. In this pipeline, a default acceptance threshold (user-configurable) of ≤6 Å is used. This value serves as a conservative quality gate, rejecting only micrographs with clearly compromised high-resolution content.
(ii) *Defocus range*. The mean defocus is constrained within a user-defined range (default 1000–40 000 Å) to enforce consistency with the nominal defocus values set during data acquisition. Micrographs acquired at very high defocus values offer stronger contrast but typically lack recoverable high-resolution information, whereas those acquired at very low defocus values may contain high-resolution signal but suffer from insufficient contrast for reliable particle detection and CTF estimation. Enforcing a broad, acquisition-consistent defocus range primarily serves as a sanity check to identify and remove outliers indicative of acquisition or estimation failures, rather than as a fine-grained optimization criterion.
(iii) *Normalized astigmatism ratio*. Astigmatism, caused by lens asymmetry, results in different defocus values along two perpendicular axes, introducing directional blurring. Filtering based on the absolute defocus difference alone is suboptimal, as its severity depends on the overall defocus magnitude. A fixed difference is much more detrimental at low defocus than at high defocus. To account for this, we use a normalized astigmatism ratio: 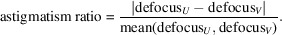
Micrographs with a ratio exceeding a user-defined threshold (default 0.1) are discarded. This threshold was chosen as a conservative cutoff to exclude micrographs with pronounced directional aberrations while retaining those with mild, typically correctable astigmatism. Examples of an accepted and a rejected micrograph using this criterion are provided in Supplementary Fig. S5.

All default thresholds reported above were derived from prior experience and empirical benchmarking across multiple datasets and acquisition conditions. They are intended to serve as generally applicable quality gates rather than being optimized for any specific dataset.

This program is integrated into the *Xmipp* software plugin as the CTF consensus protocol. Although used in this pipeline as a CTF quality filter, it can also be used to compare multiple CTF estimations and compute a consensus resolution. The algorithm assumes that two CTF estimates are consistent if their phases (wave aberration functions) differ by less than 90°. The reported consensus resolution corresponds to the spatial frequency at which the phase difference between the two CTFs reaches 90°.

### 2.3. Automated particle picking and model training

Accurate particle identification is essential for high-resolution cryo-EM reconstruction, but it remains challenging due to the extremely low signal-to-noise ratio (SNR) of cryo-EM micrographs, which makes particles difficult to distinguish from background noise, ice contamination and artifacts (Dhakal *et al.*, 2023 ▸). Numerous particle-picking approaches exist, from traditional template-matching to modern machine-learning (ML) methods such as *crYOLO* and *Topaz* (Wagner *et al.*, 2019 ▸; Bepler *et al.*, 2019 ▸). Despite these advances, generalization remains a persistent challenge. Models trained on previously characterized proteins may perform poorly on new datasets with different particle sizes, shapes or imaging conditions (Chen *et al.*, 2025 ▸). This limitation underscores the need for automated strategies that adapt to the specific characteristics of each dataset without manual intervention.

**Figure 3:**
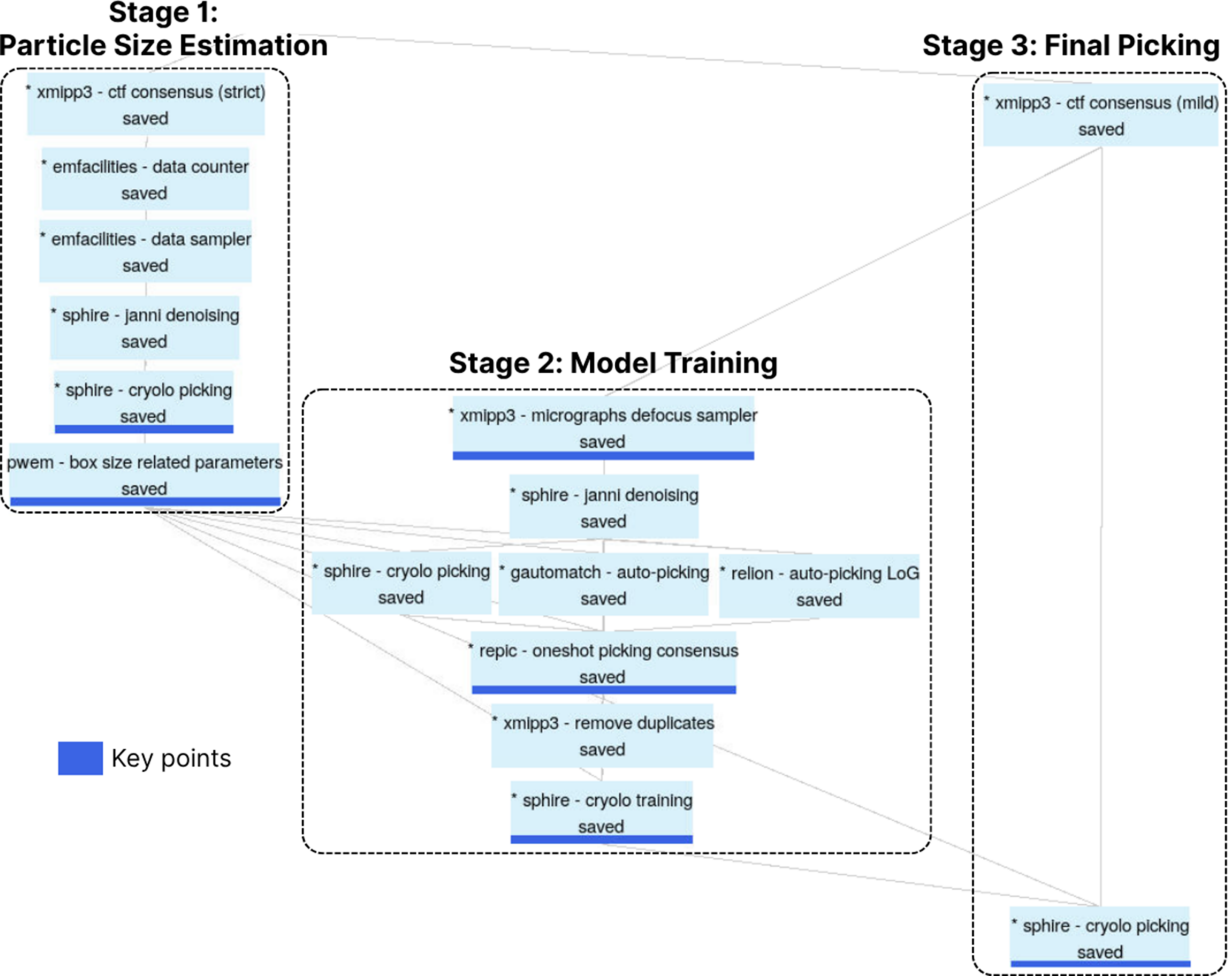
Detailed *Scipion* workflow diagram for the automated particle-picking strategy. The strategy begins with curated micrographs from early preprocessing and is divided into three stages: Stage 1, automatic particle size estimation; Stage 2, consensus-based ground-truth generation and model training; Stage 3, final picking with the data-specific model. Dark blue labels indicate the key protocols for the automated picking strategy.

A central innovation of our workflow is the on-the-fly training of a data-specific particle-picking model. The goal is to automatically generate a reliable training dataset and use it to train a particle-picking model tailored to the imaged sample. The process is divided into three stages (Fig. 3 ▸).

#### 2.3.1. Stage 1: automatic particle size estimation

A key prerequisite for most pickers is particle diameter, which is often unknown for novel proteins. To automate its estimation, the workflow selects a small subset of high-quality micrographs: from the first 100 curated micrographs that pass a stringent CTF resolution filter (<5 Å) and are within a moderate-to-high defocus range (10 000–30 000 Å) to ensure sufficient particle contrast, eight are randomly chosen. To enhance particle visibility, these are denoised using *JANNI*, a neural-network-based denoiser designed for low-SNR cryo-EM data (Wagner & Raunser, 2020 ▸). The denoised micrographs are then passed to *crYOLO*’s internal box-size estimator for a reliable diameter estimate.

Because particle diameter determines picking box size, an inaccurate estimate can negatively affect downstream processing steps. To mitigate this risk, the workflow allows user intervention at any stage of execution. As described in Section 2.6, processing can be paused and resumed from intermediate steps without repeating earlier computations. This flexibility enables users to adjust the estimated particle diameter and restart the picking stage if necessary. Additionally, the particle size can be explicitly defined within the workflow template, allowing experienced users to override the automatic estimation when prior knowledge about the sample is available.

#### 2.3.2. Stage 2: consensus-based ground-truth generation and model training

After estimating the particle diameter, the workflow generates a robust set of ground-truth particle coordinates for training. To capture variability across defocus levels, a custom program performs defocus-balanced sampling: from the first 250 curated micrographs, 25 spanning the full defocus range are selected and denoised with *JANNI* for initial picking.

Using the estimated particle diameter, three template-free picking algorithms are then automatically configured and executed on the denoised micrographs: the neural-network-based picker *crYOLO* (Wagner *et al.*, 2019 ▸), the correlation-based *Gautomatch* (K. Zhang, M. Li & F. Sun, unpublished work) and *RELION*’s Laplacian-of-Gaussian (LoG) picker (Kimanius *et al.*, 2021 ▸). Particle picking remains a challenging task in part because no single algorithm performs optimally across all samples. Each method is trained on different datasets and/or encodes its own assumptions about what constitutes a particle, leading to variation in how signal and background noise are distinguished. Consequently, the performance of any given picker cannot be reliably predicted for a new or difficult specimen (Cameron *et al.*, 2024 ▸). Combining the outputs of multiple pickers with different underlying algorithms helps to generate a broader and more representative set of initial particle candidates.

*Gautomatch* and the *RELION* LoG picker, although both blob-based, rely on different mathematical formulations and parameterizations, resulting in distinct candidate sets. *Gautomatch* remains widely used despite lacking active maintenance, and *Scipion* manages legacy dependency issues by encapsulating required toolkits and binaries in a conda environment. Furthermore, the modular design of the workflow enables the straightforward integration of additional or alternative pickers, such as deep-learning-based approaches such as *Topaz*, as they become available.

The three pickers’ coordinates are compared using *REPIC* (Cameron *et al.*, 2024 ▸). *REPIC* frames the consensus problem as an integer linear programming optimization task that identifies particles consistently detected across multiple pickers. This approach reliably generates high-quality training data when the optimal picker is unknown. *REPIC*’s consensus set typically matches the best-performing individual picker while remaining robust to poor performance by any single method, effectively automating what was previously a manual step (Cameron *et al.*, 2024 ▸).

The resulting consensus coordinates serve as ground truth to train a new *crYOLO* model via transfer learning from the general *crYOLO* model, thereby accelerating the process. Training uses the 25 original (non-denoised) micrographs with coordinates from the denoised images, benefiting from the improved picking performance on denoised images while avoiding the computational cost of denoising the entire dataset. Additionally, *crYOLO*’s tolerance for sparse labels makes it well suited for automated training (Wagner *et al.*, 2019 ▸).

This consensus-based ground-truth generation and transfer-learning approach enables the fully automated creation of a dataset-specific particle-picking model, eliminating the need for manual annotation.

#### 2.3.3. Stage 3: final picking with the data-specific model

Once trained, the dataset-specific *crYOLO* model is applied to all curated micrographs from the data-collection session. Fine-tuning via transfer learning has distinct advantages over directly applying the general model: the latter, while trained on diverse datasets, may not optimally capture the unique characteristics (size, contrast, orientation, ice thickness, contamination) of new samples.

By initializing with the pretrained general model and fine-tuning with data-specific particles, the workflow adapts to unique imaging conditions and sample features. This strategy combines broad feature recognition with dataset-specific refinement, improving discrimination between particles and background while requiring only minimal automatically generated training data. The resulting model achieves high precision and recall, producing quality coordinates for downstream 3D reconstruction.

This improvement is illustrated in Supplementary Fig. S6, which presents both a qualitative comparison of picking behavior and a quantitative evaluation via downstream 2D classification. The results demonstrate the improved selectivity and precision of the data-specific model.

### 2.4. Initial 2D and 3D analysis

**Figure 4:**
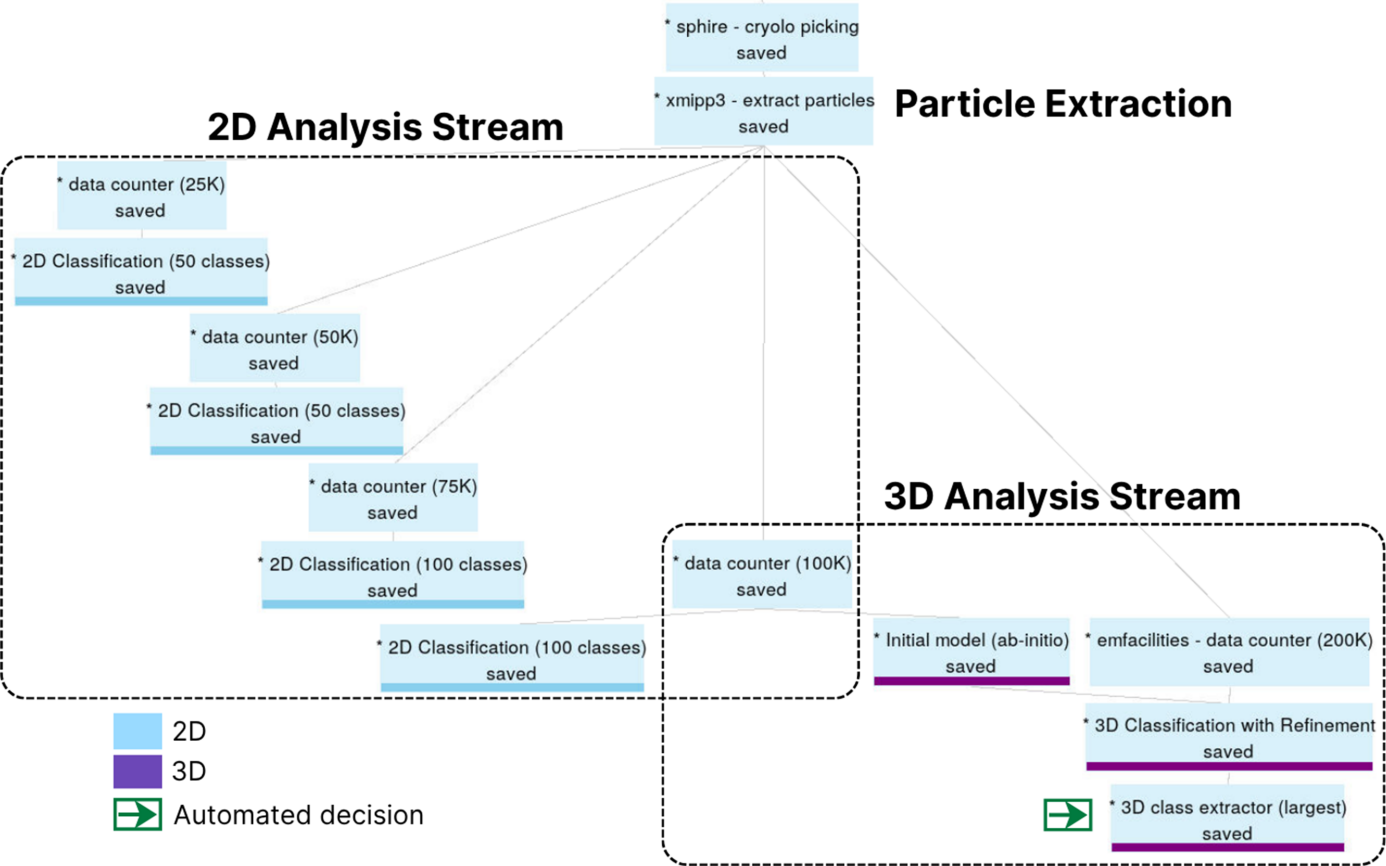
Detailed *Scipion* workflow diagram for the initial 2D and 3D analysis stage. After particles are picked with the data-specific picking model and extracted, the stream of particles is analyzed in two concurrent analysis streams: sky-blue labels correspond to image-processing steps in the 2D analysis stream, while purple labels denote steps in the 3D analysis stream. Automated decisions are indicated with green labels, culminating in the selection of the 3D class with the largest population of particles.

Following particle picking, the pipeline enters the third stage: initial 2D and 3D analysis (Fig. 4 ▸). After particle extraction, the workflow splits into two parallel processing streams. One performs consecutive 2D classification to generate updated class averages, offering a dynamic view of particle quality as data accumulates. The other stream generates two *de novo* 3D models and uses them for an initial 3D classification to separate true particles from noise and contaminants. This dual approach delivers rapid, comprehensive structural feedback during data collection.

#### 2.4.1. Particle extraction

Particles are extracted from curated micrographs using the *Xmipp* particle-extraction protocol. The extraction box is set to twice the picking box size to capture the delocalized CTF signal. Contrast is inverted, and particles are normalized to zero mean and unit variance. Particles near the micrograph borders are removed, and a dust-removal filter eliminates pixels with abnormally high values. To optimize for speed and memory, particles are downscaled to a standard 128 × 128 pixel box, which is sufficient for initial, lower-resolution analysis while drastically reducing computational load.

#### 2.4.2. 2D analysis stream

The 2D analysis stream provides a continuously updated view of sample quality. It begins after 25 000 particles are extracted and repeats cumulatively with each new batch, up to a maximum of 100 000 particles. In the standard workflow, 2D classification is performed sequentially on batches of 25 000, 50 000, 75 000 and 100 000 particles. This iterative 2D classification uses a multi-reference alignment (MRA) approach. The workflow supports both the robust expectation–maximization algorithm in *RELION *(Kimanius *et al.*, 2021 ▸) and the faster stochastic gradient-descent (SGD) method in *Cryo­SPARC* (Punjani *et al.*, 2017 ▸), depending on software availability and licensing. This design enables continuous assessments of particle views and sample homogeneity throughout data collection. An example of 2D class average evolution with cumulative batches is provided in Supplementary Fig. S7.

Batch size and maximum particle limit are user-configurable; larger cumulative batches (*e.g.* 50 000) can be used if desired. Incrementing by 25 000 balances computational efficiency and the ability to observe improvements in class averages. The workflow is also easily extendable: additional 2D classification branches can be added by duplicating branches and increasing the particle-count threshold, allowing full-dataset classification if needed. If data collection ends before reaching the set particle limit, a final 2D classification is performed using all available particles. Because each classification job requires GPU resources, users should balance batch size and number of jobs for optimal computational efficiency.

#### 2.4.3. 3D analysis stream

In parallel, the 3D analysis stream generates two initial 3D structures and separates true particles from noise and contaminants. The process is hierarchical and uses a substantial subset of particles.

(i) *Initial model generation*. The first subset of up to 100 000 particles is used to generate two independent *de novo* 3D models using an SGD-based approach in either *RELION* or *CryoSPARC*. This is performed without symmetry or external templates to avoid bias.
(ii) *3D classification with refinement*. These two *ab initio* models are then used as references for 3D classification of a larger subset (up to 200 000 particles). This critical step separates well behaved particles that align consistently with a structured reference from non-particles or ‘junk’ that align poorly.

Most datasets processed with this workflow exhibit a clear separation: one 3D class contains structured, protein-like features that improve with refinement, while another converges toward low-resolution, blob-like density representing noise or poorly aligned particles. Assuming effective upstream picking, the 3D class with the largest particle population is automatically selected, as it typically represents the most prevalent and stable state of the macromolecule. In these cases, selecting the most populated class is an effective automated strategy for isolating true particles and yields a high-purity particle set for downstream refinement. Examples of 3D classification supporting this approach are provided in Supplementary Fig. S8.

It is uncommon for particles to split into two biologically relevant conformational states at this stage. The main goal here is not to resolve structural heterogeneity, but to quickly separate true particles from artifacts. However, if early separation into distinct conformations occurs, it can already provide valuable insight into conformational variability; in such cases, the workflow automatically retains the most populated and well resolved class. Comprehensive analysis of conformational heterogeneity is deferred to subsequent 3D analysis stages (Section 2.5.2), where a cleaner set of true particles is available.

The number of particles for each processing task is user-configurable, allowing users to expand the analysis to larger particle sets if desired. We recommend keeping the *ab initio* model-generation step at approximately 100 000 particles, as this is typically sufficient to produce at least one protein-like reconstruction. For the 3D classification with refinement step, increasing the number of particles may improve the resolution achievable in subsequent stages, as larger particle sets generally yield higher resolution reconstructions. However, this also increases the computational cost.

### 2.5. Refinement and parallel validation

**Figure 5:**
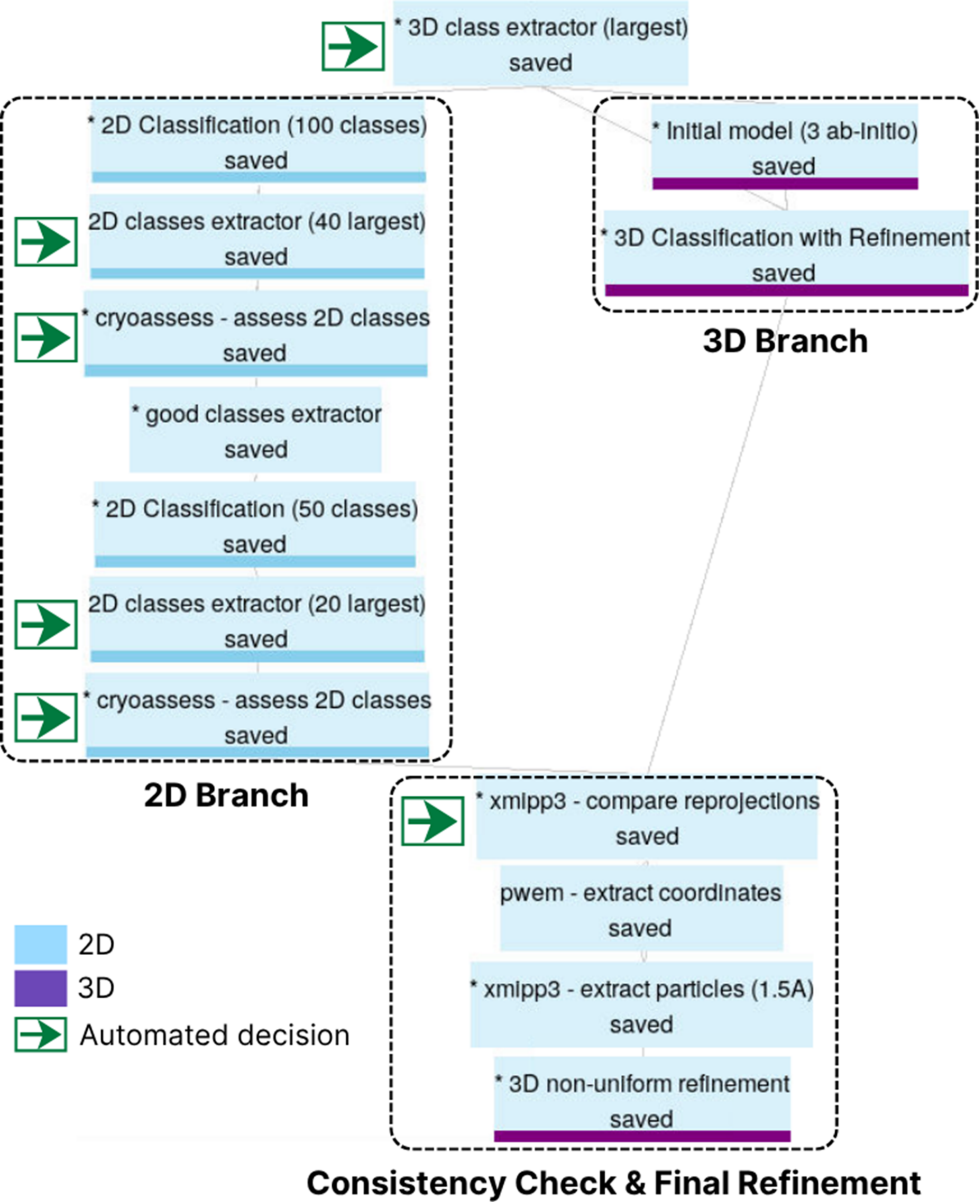
Detailed *Scipion* workflow diagram for the refinement and parallel validation stage. After pruning particles during early 3D classification, particles from the most populated class are analyzed in two concurrent processing branches: the 2D branch and the 3D branch. Results from both branches are cross-validated (consistency check) to select the optimal particle set for final refinement. Sky-blue labels correspond to image-processing steps for the 2D branch, while purple labels denote those for the 3D branch. Automated decisions are indicated with green labels, highlighting critical steps for this stage.

The fourth and final stage of the pipeline is designed to uncover sample heterogeneity, produce a high-quality particle set and generate a refined 3D reconstruction. This stage employs a parallel validation strategy: the filtered particle set is split into two independent branches: one for consecutive 2D classification and one for 3D classification. Results from both branches are cross-validated to select the optimal particle set for final high-resolution refinement (Fig. 5 ▸).

#### 2.5.1. 2D branch: consecutive classification and selection

The goal of the 2D branch is to obtain a high-resolution set of 2D class averages using a two-round strategy that combines classification with automated, deep-learning-based selection.

(i) *First round of classification and selection*. The input particles are classified into 100 2D classes using exhaustive search. A two-step automated selection follows.
  (1) The 40 most populous classes are selected.
  (2) These 40 classes are evaluated by 2*D-Assess*, a deep-learning tool for automated 2D class average assessment (Li, Cash *et al.*, 2020 ▸). Only particles from classes labeled as ‘good’ are retained.
  This hybrid approach, combining population-based pre-filtering with a deep-learning selector, is more robust than single-method approaches and reduces the likelihood of selecting false positives.
(ii) *Second round of classification and selection*. The curated particles then undergo a second, more refined 2D classification into 50 classes. The algorithm’s uncertainty factor is increased to better separate subtly different particle views. The same two-step selection is applied: the 20 most populous classes are chosen and 2*D-Assess* is used to select only the ‘good’ classes from this subset. This results in a high-resolution set of 2D class averages.

An example of this automated decision-making process is shown in Supplementary Fig. S9, illustrating both rounds of classification and how the combined population-based and deep-learning selection criteria progressively refine the dataset to yield higher quality 2D class averages.

#### 2.5.2. 3D branch: heterogeneity analysis

In parallel, the 3D branch generates refined 3D classes to separate particles based on conformational or compositional differences. Three independent *de novo* 3D models are generated, serving as references for an exhaustive 3D classification with refinement. This step rigorously removes remaining noise and heterogeneity, with the goal that at least one class converges to a high-resolution structure representing the main particle state.

#### 2.5.3. Consistency check: cross-validation of 2D and 3D results

This parallel structure enables a robust self-consistency check: high-resolution 2D class averages serve as independent ground truth to objectively rank and select the best 3D class from the 3D branch. For each 3D class, a complete set of 2D projections is generated. For each ‘good’ 2D class average, an exhaustive search identifies the best-matching projection from each 3D volume and a cross-correlation score is calculated. The mean of these scores reflects how well each 3D volume agrees with the 2D data. High-resolution features in the 2D averages will only correlate well with projections from a 3D volume that also contains those features. The 3D volume with the highest mean correlation score is selected as the best structural representation. An example of consistency check results for cross-validating the 2D and 3D results is provided in Supplementary Fig. S10, clearly demonstrating that the best-ranked volume was the correct choice for final 3D refinement. This ranking is performed automatically by an updated version of the *Xmipp* compare reprojections protocol.

#### 2.5.4. Final 3D refinement

With an optimal particle set and reliable 3D model from the consistency check step, the pipeline performs final refinement to yield a preliminary high-resolution reconstruction before data acquisition concludes.

(i) *Re-extraction of particles at higher resolution*. To recover high-frequency information lost during initial downsampling, curated particles are re-extracted from the original micrographs at a larger box size, corresponding to a pixel size of 1.5 Å per pixel. This balances high-resolution detail with the computational speed needed for on-the-fly feedback.
(ii) *3D refinement*. A high-resolution non-uniform refinement is performed (using *CryoSPARC* or *RELION*) on the re-extracted particles. Iteration continues until the 3D map converges. Final resolution is estimated by Fourier shell correlation (FSC) between two independently refined half-maps. Per-particle CTF refinement is not performed at this stage to reduce computational cost and avoid overfitting during early reconstruction. It is recommended as a separate post-processing step for a final structure.

### 2.6. Pipeline execution model

*Scipion* templates enable complete workflows to be loaded and executed in a single step, supporting the deployment of full image-processing pipelines. These templates accept user-defined *dynamic parameters* that can be adjusted at execution time via the graphical interface or the command line, enabling fully unattended operation. Dynamic parameters may include acquisition settings (*e.g.* movie paths, microscope voltage, magnification, pixel size) and processing parameters (*e.g.* CTF resolution limits, particle size, number of particles for initial 2D/3D analyses). This design offers flexibility in workflow selection and allows users to specify which parameters are fixed or adjustable for each experiment.

To ensure broad applicability, default templates and thresholds are robust for standard, untilted single-particle acquisition, regardless of the microscope model. In this scenario, users typically only specify acquisition parameters. For specialized cases such as tilted data collection, dedicated templates incorporate relaxed motion-correction thresholds (per-frame shift = 30 Å; global drift = 120 Å), relaxed CTF filtering criteria (resolution cutoffs of 6.5 and 8.5 Å) and an expanded defocus-sampling pool (500 micrographs to select 25 training examples) to improve model generalization. These settings are empirically derived from multiple datasets to provide robust defaults rather than dataset-specific tuning.

The pipeline operates in streaming mode: downstream protocols are triggered as soon as the required inputs become available, enabling true on-the-fly processing and continuous data-quality feedback. For robustness, the workflow incorporates fault-tolerance mechanisms. Streaming preprocessing steps (*e.g.* motion correction, CTF estimation, particle picking) implement per-job fault tolerance: if an image or batch fails due to transient issues (I/O errors, timeouts, resource unavailability), it is skipped and processing proceeds with subsequent inputs.

For protocols operating on aggregated datasets in a single processing step (*e.g.* 2D or 3D classification), recovery is achieved through manual restart mechanisms. *Scipion* also allows users to stop, restart or duplicate protocols at any point during execution, enabling intervention without recomputing upstream results. For instance, if low-quality micrographs are detected but exploratory 2D classification is still needed, users can stop at the CTF filter step, adjust the resolution limit and resume processing from that point onward.

The minimum hardware requirement for on-the-fly workflow operation is four GPUs. Two are dedicated to motion correction (the main preprocessing bottleneck), with the remaining two shared across the other steps. For stability, the workflow is typically managed by a job scheduler (*e.g.SLURM*) to prevent multiple jobs from competing for the same GPU and to avoid memory exhaustion. In this configuration, preprocessing matches the current data-acquisition rate. However, steps such as *Miffi* filtering and particle picking share GPU resources with longer tasks (*e.g.* 2D classification, *ab initio* reconstruction), which may cause delays. For uninterrupted on-the-fly feedback, including 2D and 3D analysis, at least five or six GPUs are recommended to allow intensive steps to run without delaying preprocessing.

The modular design of *Scipion* templates enables execution of reduced workflows in resource-limited environments. Users can run only preprocessing or include intermediate steps, such as 2D classification, while deferring 3D processing by selecting simpler pipeline versions. Workflow templates of varying complexity, from lightweight preprocessing to full 3D reconstruction, are available via the WorkflowHub collection ‘CryoEM Facility Workflows’ (Scipion CNB, 2025 ▸), allowing facilities to tailor execution to their computational capacity.

Currently, the pipeline does not automatically terminate data acquisition. Quality-control protocols monitor and flag issues, but decisions on whether to continue or stop data collection are left to the user. Similarly, the pipeline does not dynamically tune parameters based on data quality; instead, it uses empirically validated default thresholds as quality filters to prevent severely degraded data from propagating downstream.

## 3. Results

The following sections present the results of applying this pipeline across a wide range of scenarios, from extensive benchmark datasets (Dhakal *et al.*, 2023 ▸) to its real-world deployment as the standard processing solution at the high-throughput cryo-EM facility of the European Synchrotron Radiation Facility (ESRF). The workflow templates used in this study are publicly available in the WorkflowHub collection called ‘CryoEM Facility Workflows’ (Scipion CNB, 2025 ▸), which also provides detailed documentation. This includes two documents: (i) a manual for using *Scipion* in the context of a cryo-EM facility, focusing on three main areas, on-the-fly processing, workflows and template design, and integration with queue systems; and (ii) a more specific guide describing the available templates, image-processing details, implementation aspects and required software. WorkflowHub itself is a community registry for computational workflows (Gustafsson *et al.*, 2025 ▸).

### 3.1. Overall workflow outcome

Upon completion of the acquisition session, the automated pipeline concludes, providing the user with a comprehensive suite of processed data and quality-control metrics. This organized output serves as a robust foundation for subsequent, more detailed post-acquisition analysis. The key deliverables include the following.

(i) A complete set of aligned movies and their corresponding CTF estimations.
(ii) A curated set of high-quality micrographs accompanied by detailed quality measurements.
(iii) A data-specific particle-picking model trained on the user’s sample.
(iv) A complete set of picked-particle coordinates from all curated micrographs.
(v) High-quality 2D class averages representing the particle’s different views.
(vi) The best 3D volume from the parallel validation stage, along with its associated high-purity particle set.
(vii) A preliminary high-resolution 3D structure.
(viii) A complete record of all processing steps and their outcomes, providing critical feedback to guide further, in-depth image processing (*Scipion*’s project).

### 3.2. Extensive benchmark

To validate the pipeline’s robustness and general applicability, we performed an extensive benchmark on the CryoPPP dataset (Dhakal *et al.*, 2023 ▸). This dataset, derived from the Electron Microscopy Public Image Archive (EMPIAR), comprises 32 nonredundant, diverse protein targets that vary significantly in terms of size, molecular weight, symmetry and sample characteristics. While the original CryoPPP dataset limits entries to approximately 300 micrographs, this number is often insufficient to yield enough particles for a meaningful 3D reconstruction. To create a test scenario that more closely mirrors a real-world, on-the-fly processing session, we expanded our test set by selecting up to 1000 micrographs for each entry, ordered by filename in ascending order. This standardized subset size ensures sufficient data for a robust 3D reconstruction attempt while reinforcing the pipeline’s diagnostic goal: to determine sample viability from a representative fraction of the data during acquisition, rather than requiring a complete, multi-terabyte dataset.

Although the datasets were downloaded in advance from EMPIAR and processing was initiated once the full datasets were locally available, all experiments were executed using *Scipion*’s streaming execution model. That is, the full workflow was launched once, and downstream protocols began processing as soon as their required inputs became available from upstream steps, without waiting for the entire dataset to complete. We therefore did not simulate microscope acquisition rates (*e.g.* movies arriving every few seconds), but we preserved the internal execution behavior of the unattended, on-the-fly pipeline. This allowed us to benchmark the workflow’s robustness across diverse specimens and acquisition conditions under controlled, reproducible conditions, decoupled from network throughput and acquisition latency.

For all benchmark experiments reported in this section, processing parameters and quality-control thresholds were kept fixed across datasets. This was possible because all selected EMPIAR entries corresponded to nontilted single-particle data collections, enabling the use of a single standardized workflow template and allowing fair comparisons of pipeline behavior across heterogeneous samples without dataset-specific tuning.

A crucial aspect of this study is defining a success metric suitable for an automated diagnostic pipeline. While the gold-standard Fourier shell correlation (FSC) is indispensable for reporting the final resolution of a manually processed structure, it can be a misleading metric for success in a fully automated context. An automated pipeline may converge on nonparticle features (*e.g.* ice contaminants, carbon edges) that still produce a high-resolution FSC curve, leading to a false-positive result. Conversely, a pipeline may correctly identify a challenging biological sample. Still, inherent issues, such as conformational heterogeneity or flexibility, that require expert manual intervention to resolve may limit achievable resolution, leading to a false-negative assessment of the pipeline’s performance if judged solely by a resolution threshold.

Therefore, we defined the primary success criterion as the visual inspection of the final 3D reconstruction. A test case was considered successful when the resulting 3D map exhibited recognizable, protein-like features consistent with the expected size and shape of the target macromolecule. This qualitative metric was chosen because it directly addresses the central question of whether the workflow can robustly handle the complexity of diverse specimens and acquisition conditions, providing a more meaningful measure of its value as a diagnostic tool for assessing sample quality and the feasibility of high-resolution structure determination.

This benchmark was performed on a CentOS 7 Linux server with 40 cores (2× Intel Xeon Gold 6230, 2.20 GHz) and 384 GB of RAM. The workstation also featured four GPUs (Tesla T4 Driver Version 460.27.04, CUDA Version 11.2) with 16 GB each. In terms of storage, it has four 8 TB SATA HDDs in a RAID 5 configuration for mass storage (where data was stored), two 1 TB SATA SSDs in a RAID 0 configuration for scratch and two 240 GB SATA SSDs. This machine is housed within the Biocomputing Unit data center at the Spanish National Centre for Biotechnology (CNB–CSIC). It is important to note that *CryoSPARC* was used through the *Scipion* framework, via the *scipion-em-cryosparc* plugin, as the processing backend for the 2D and 3D steps.

As shown in Table 1 ▸, the pipeline successfully processed the majority of datasets (94%) while adapting to significant variations in particle distribution, noise, defocus and sample conditions without manual tuning. In 78% of the samples, the workflow achieved robust 3D reconstruction, revealing the protein’s symmetry and proper shape, and even reached the Nyquist limit of 3 Å in some reconstructions (all particles were extracted at 1.5 Å per pixel). In the other 16% of cases tagged as successful, even when the studied particles were present, the final 3D reconstruction was not optimal, lacking high-resolution details or specific particle orientations. For the entries that did not yield a structure (6%), a more in-depth analysis was performed to determine whether the problem lay with the processing or was inherent to the dataset’s complexity.

To demonstrate the robustness of the diagnostic automated pipeline, we selected five high-quality cases, all suboptimal cases and all failed cases for in-depth analysis of the feedback provided by our tool. The image-processing summary is shown in Table 2 ▸.

#### 3.2.1. High-quality cases

**Figure 6:**
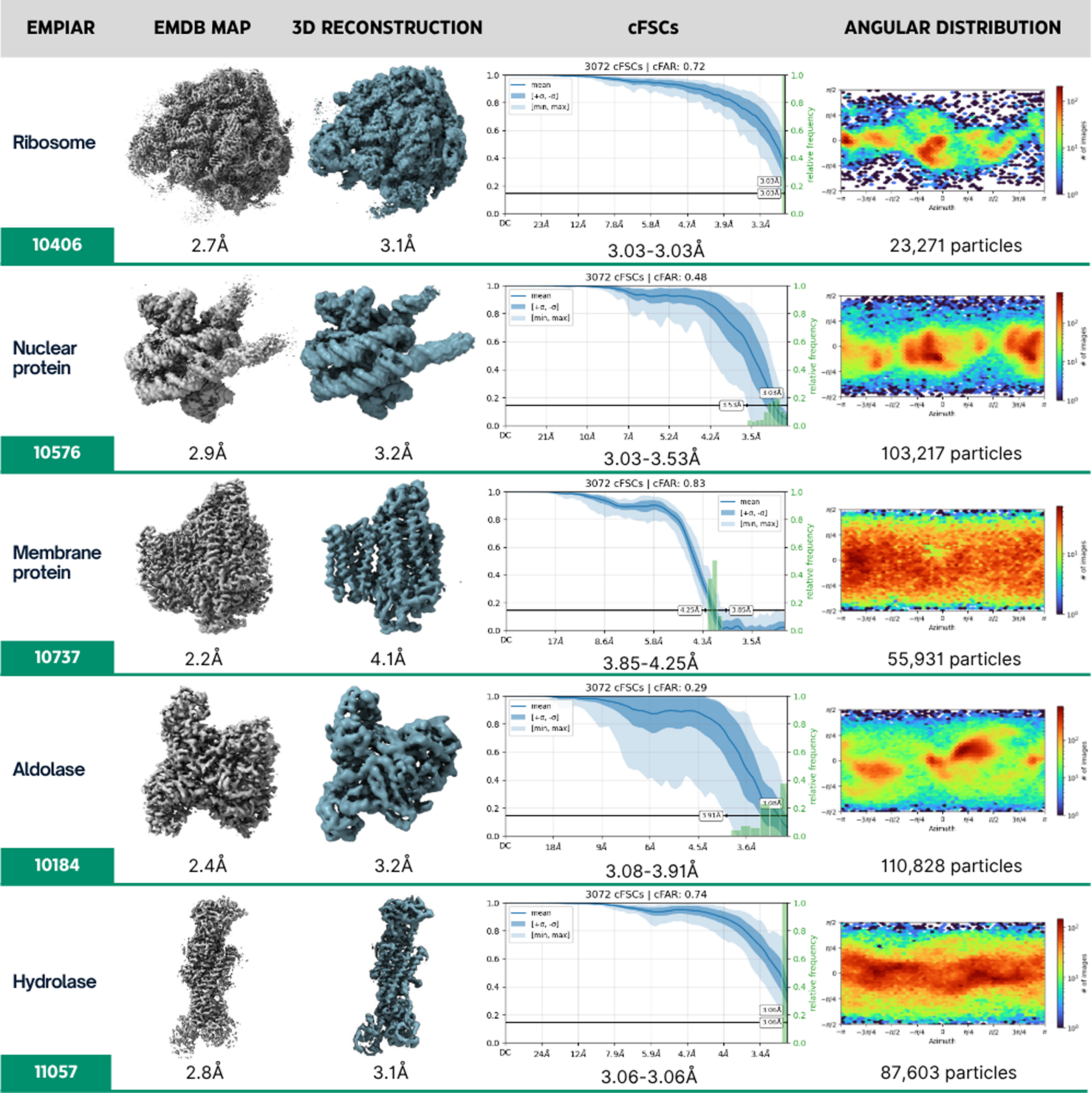
High-quality examples: each entry corresponds to one of five distinct proteins with unique shapes and molecular weights. For each case, the deposited EMDB volume, reconstructed 3D map (raw, unsharpened reconstruction) and key quality metrics are shown: conical Fourier shell correlation (cFSC) plots and particle angular distributions. The cFSC plots summarize directional resolution anisotropy; ideally, narrow standard deviation bands around the mean indicate isotropic resolution and uniform directional quality. The orientation distribution plots reveal angular coverage: uniform coverage indicates well sampled orientations, while anisotropy or clustering reveals preferred orientations that can limit achievable resolution.

For the high-quality cases, five of 25 very different proteins were carefully selected to show the ability of the workflow to adapt to other conditions, shapes, sizes, compositions and particular challenges of these EMPIAR entries (Fig. 6 ▸):

For the 70S ribosome dataset (EMPIAR-10406), our workflow was challenged with a very large ribonucleoprotein particle composed of ribosomal RNA and numerous protein subunits. This system is not only a widely used benchmark in cryo-EM but is also a representative of highly heterogeneous and asymmetric macromolecular assemblies. Despite processing only 36.8% of the deposited data, the workflow successfully reconstructed the conformation corresponding to EMDB entry EMD-10809. It even detected the alternative state reported as EMDB entry EMD-10892 during the first round of 3D classification, which was then excluded from subsequent steps. This demonstrates that the pipeline can resolve conformational heterogeneity without requiring manual intervention. Importantly, automated curation retained only 58.8% of the micrographs, with most rejections flagged by *Miffi* as bad films, in agreement with the dataset’s known issues of moderate protein edge texture. These results highlight the pipeline’s ability to robustly process large, complex and heterogeneous assemblies while also informing users about data-quality issues.

The nucleosome dataset (EMPIAR-10576) provided a distinct test case consisting of a DNA–protein complex of moderate size with a characteristic elongated helical structure of DNA wrapped around histone proteins. Unlike compact globular proteins, nucleosomes often yield micrographs with uneven contrast, in which the DNA ends contribute weaker signals than the protein core, resulting in the ends appearing blurred in 2D averages. Despite these particularities, and using only 50% of the deposited micrographs, the workflow achieved reliable particle picking, with 2D classes that clearly captured the DNA-wrapped contours that became more defined after refined 3D alignment. Data curation was highly effective, with 97.4% of the first 1000 micrographs retained, confirming the dataset’s overall quality. This case demonstrates the pipeline’s adaptability in handling nucleic acid–protein complexes, producing high-quality 2D classes and 3D reconstructions without requiring manual intervention.

The cytochrome *bo*_3_ dataset in MSP nanodiscs (EMPIAR-10737) represented a third distinct scenario, testing the pipeline on a membrane protein embedded in a lipid bilayer mimic. The lipid nanodisc introduces additional background, complicating particle picking, as empty nanodiscs or lipid belts can dominate early classifications. Even with only 29% of the deposited micrographs, the workflow effectively identified the protein signal. After the initial 3D classification, the nanodisc boundaries were evident in the classes. However, subsequent classification steps minimized the lipid background, ensuring that the reconstruction focused on the protein core. This resulted in the recovery of high-resolution features in the membrane protein itself. Automated data curation accepted 75.2% of the initial 1000 micrographs, ensuring that only the highest quality data progressed to processing. This example highlights how the pipeline addresses the unique challenges of membrane proteins, where empty lipid signals must be distinguished from protein density.

The aldolase dataset (EMPIAR-10184) served as an example of a small, symmetric and well behaved soluble enzyme. Its high particle count and intrinsic symmetry make it an ideal benchmark for testing throughput, efficiency and resolution. In this case, the workflow processed 62% of the deposited data using *C*1 symmetry, yet still recovered symmetric features and achieved an almost Nyquist-limited reconstruction, despite the particles being extracted at 1.5 Å resolution in all cases. Data curation retained only 47.5% of the first 1000 micrographs, effectively discarding those with low resolution or empty fields, illustrating the ability of automated filtering to clean the dataset. This case demonstrates the pipeline’s efficiency and reliability under sub­optimal conditions, showing that it can achieve near-maximum resolution automatically without manual supervision.

Finally, the hydrolase dataset (EMPIAR-11057) provided another contrasting test case, consisting of a gastric proton pump complexed with revaprazan, a relatively small membrane protein (149 kDa), solubilized in detergent with a nonsymmetric, cylindrical shape. This sample was notoriously difficult to process due to its elongated geometry, small size, relatively low contrast and a micelle coating its transmembrane region, which complicates particle picking and alignment. Moreover, this dataset was labeled as suboptimal due to difficulties in particle identification, low particle concentration and ice contamination. Despite these challenges, and using only 12% of the deposited micrographs, the workflow still performed reliable particle picking. However, some false positives in empty micrographs were also detected in early 2D classes. Automated data curation was effective, with 80.4% of the first 1000 micrographs accepted, showing that the system could still distinguish usable data under challenging conditions. This case highlights the robustness of the pipeline in handling weak-signal particles and irregularly elongated shapes.

#### 3.2.2. Suboptimal cases

**Figure 7:**
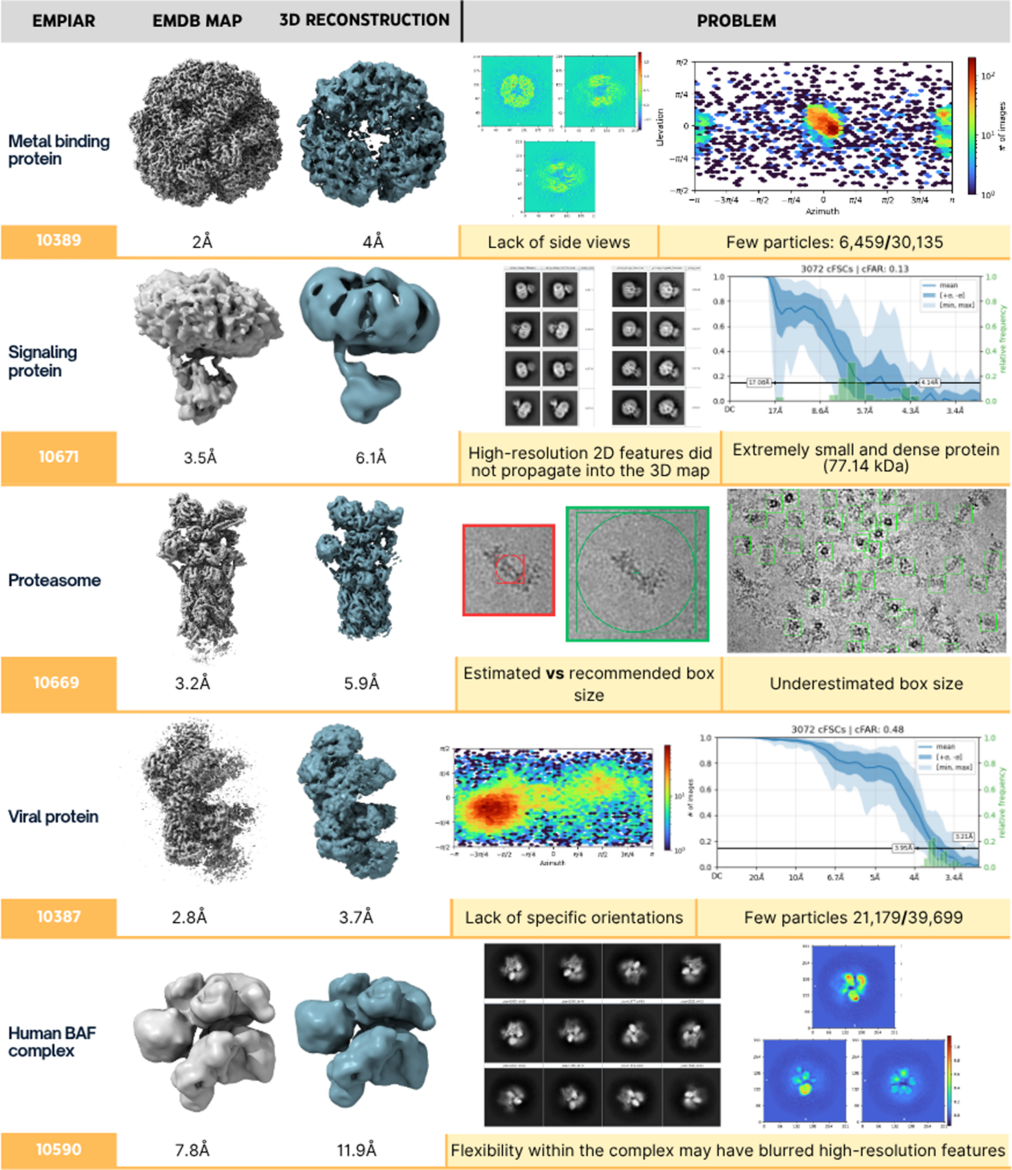
Suboptimal cases: each entry corresponds to one of five datasets in which the final 3D reconstruction was classified as suboptimal. For each case, the deposited EMDB volume, the reconstructed 3D map (raw, unsharpened reconstruction) and the specific issues identified by the pipeline are shown, highlighting the reasons for suboptimal reconstruction quality.

For the suboptimal cases, reviewing the image-processing results helps us identify why the 3D reconstruction was not optimal (Fig. 7 ▸). Nevertheless, it is essential to note that in all five cases the target protein was still visible in the reconstructions.

For the metal-binding protein dataset (EMPIAR-10389), corresponding to urease from *Yersinia enterocolitica*, our workflow successfully identified the target protein; however, the final 3D reconstruction lacked side views. This limitation is likely related to the relatively small number of initial particles (30 135), the use of only 23.1% of the deposited micrographs and a strong pruning effect during automated data curation, which retained only 65.4% of the first 1000 images. The dataset itself is annotated as being affected by abundant ice patches, dispersed particles and low particle density per micrograph, and our results corroborated these issues. A potential improvement for future processing would be to increase the number of initial micrographs, thereby boosting particle counts and sampling missing orientations.

The signaling protein dataset (EMPIAR-10671), corresponding to the CGRP receptor bound to peptide in detergent micelles, yielded a low-resolution 3D reconstruction. Although the protein was clearly visible and recognizable in 2D classes, high-resolution features failed to propagate into the 3D map. The nature of the dataset partly explains this: the target is a very small and compact 77 kDa protein embedded in detergent micelles, which obscure structural detail. While we respected our 200 000-particle threshold, typically sufficient to reach high resolution, the presence of the micelle has limited resolution. In this case, additional manual intervention, such as masking the micelle or carefully filtering particle sets during 2D classification, could improve outcomes.

The proteasome dataset (EMPIAR-10669), corresponding to the substrate-engaged 26S proteasome, highlighted the challenges of working with elongated complexes. The reconstruction problem stemmed from an underestimation of the box size. Due to its very long cylindrical shape, side views extended beyond the extraction box and were truncated in the final 3D map. While the protein was clearly recognizable, the whole complex was not reconstructed. This issue emphasizes the need for box-size adaptation when processing very elongated particles; increasing the box dimensions would likely recover the missing density and enable a more complete reconstruction.

For the viral protein dataset (EMPIAR-10387), which investigates HIV intrasomes in complex with strand-transfer inhibitors, the reconstructed map appeared noisy with unresolved regions. Only 39 699 particles were initially picked, and processing relied on just 50% of the deposited data. Moreover, the dataset itself is annotated as containing highly aggregated particles that are difficult to pick out, a challenge that our results confirmed. Although the target complex was detectable, the scarcity of particles limited reconstruction quality. A straightforward solution would be to include a larger portion of the dataset to increase the number of particles.

Finally, the human BAF complex dataset (EMPIAR-10590), corresponding to the human BRG1/BRM-associated factor (BAF) chromatin-remodeling complex, yielded a low-resolution 3D reconstruction consistent with the deposited resolution of 7.8 Å. The overall shape matched the reference, but many finer details remained unresolved. The relatively large particle set (181 267 initial particles) suggests that insufficient sampling was not the main issue. Instead, intrinsic conformational heterogeneity and flexibility within this multi-subunit complex likely blurred high-resolution features: in the 2D classes, some regions appeared well defined while others were noticeably diffuse. In such cases, advanced heterogeneity analysis or targeted manual refinement would be necessary to overcome these limitations.

#### 3.2.3. Failed cases

**Figure 8:**
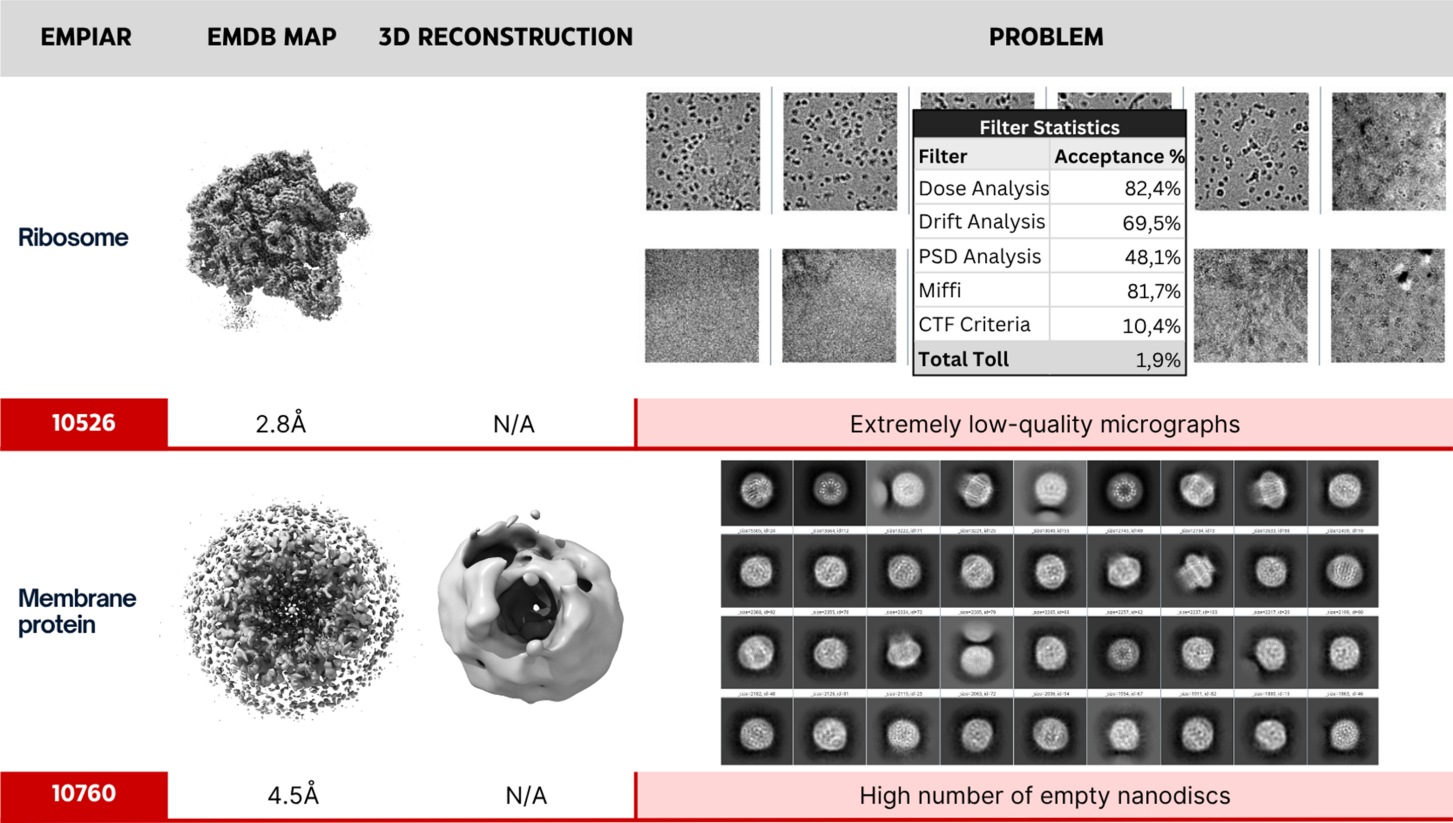
Failed cases: each entry corresponds to one of two datasets that did not yield a valid 3D structure. For each case, the deposited EMDB volume, the failed reconstructed 3D map (raw, unsharpened reconstruction) and the specific issues identified by the pipeline are shown, highlighting the reasons for reconstruction failure.

For the entries that did not yield a structure (two out of 32), it is necessary to determine whether the limitation was due to our image-processing pipeline or inherent to the dataset’s complexity (Fig. 8 ▸).

For the 50S ribosome dataset (EMPIAR-10526), the failure was not due to the data being inherently unreconstructable but rather to the strong rejection produced by the automated quality filters. Out of the 1000 micrographs processed by the pipeline, only 19 images (1.9%) passed automated curation. This dataset is annotated as containing severe ice contamination and large variations in ice thickness. To determine whether the rejection rate was due to poor data quality or overly stringent thresholds, we analyzed the behavior of the individual filters by running them in parallel rather than sequentially. As summarized in Fig. 8 ▸ (EMPIAR-10526, ‘PROBLEM’ column), the most restrictive filter was the CTF-based criterion, which accepted only 10.4% of the micrographs, followed by the PSD analysis (48.1% acceptance). When applied sequentially, as in the automated pipeline, the combined effect reduced the final acceptance rate to 1.9%.

Inspection of the CTF estimates revealed that the main rejection criterion was the resolution limit (≤6 Å), which excluded 883 micrographs. Among the rejected images, 222 exhibited extremely poor resolution estimates (850 Å), indicating clear CTF fitting failures. Visual inspection confirmed that these micrographs contained severe crystalline ice contamination and were unsuitable for downstream processing. After removing these extreme outliers, most remaining micrographs showed estimated resolutions of 6–7 Å (595 images). Further analysis indicated that many of these moderately low-resolution images were additionally rejected by other criteria, including high drift (144 micrographs with *S*_frame_ > 10 Å and *S*_movie_ > 45 Å), high defocus (209 micrographs with average defocus > 40 000 Å), PSD inconsistency (91 micrographs) and *Miffi* filtering (22 micrographs). Conversely, among the 117 micrographs that passed the resolution threshold, only 47 exhibited acceptable CTF fits, the absence of severe contamination and consistent signal. This behavior is illustrated in Supplementary Fig. S11, which shows both the distribution of CTF resolution estimates and representative examples of micrographs rejected due to severe fitting failures and contamination.

Furthermore, the PSD analysis was re-evaluated given its relatively low acceptance rate (48.1%) and the predominantly low-resolution nature of this dataset. The PSD analysis protocol relies on detecting Thon-ring consistency up to a specified resolution limit. Increasing this limit from the default 4 Å to 6 Å improved the PSD filter acceptance rate from 48.1% to 82.6%, indicating that the original configuration was overly strict due to the lack of high-resolution Thon rings. With the corrected parameter, the PSD filter primarily removed empty holes or micrographs lacking meaningful signal.

Finally, the automated pipeline was executed under relaxed filtering conditions (resolution threshold ≤ 7 Å, defocus up to 55 00 Å and PSD analysis adapted for low-resolution data), increasing the acceptance rate from 1.9% to 39.4% (394 micrographs). Under these conditions, a 3D reconstruction was obtained with an average resolution of 5.95 Å (FSC 0.143 criterion) using 8714 particles. The impact of these relaxed filtering conditions is shown in Supplementary Fig. S12, including both the set of retained micrographs and the resulting 3D reconstruction. Despite the lower data quality, the reconstruction preserves the overall structural features of the reference map, confirming that the pipeline can successfully recover the target structure when the filtering parameters are adapted to lower quality datasets.

These observations partly explain the discrepancy with the original study by Li, Pellegrino *et al.* (2020 ▸), which reported retaining 710 micrographs and obtaining a 2.8 Å resolution reconstruction using 16 182 particles. The original reconstruction used the complete dataset of 1103 micrographs rather than the 1000 used in our automated pipeline. Additionally, the original study likely involved manual inspection, iterative parameter tuning and the inclusion of micrographs with lower apparent quality that still contained usable signal. These results highlight the importance of adapting automated filtering thresholds to dataset-specific characteristics, particularly in cases where high-resolution signal is limited.

The pannexin-1 dataset in nanodiscs (EMPIAR-10760) posed a different challenge. Although particle picking was excellent, reaching the maximum limit of 200 000 particles imposed by the automated workflow, the dataset was heavily dominated by empty nanodiscs. As a result, the cumulative 2D classification produced very few classes displaying recognizable protein features. In the final cumulative batch of 2D classification (100 000 particles), fewer than 10% of the classes showed protein-like features.

During the first 3D division step, *ab initio* reconstruction yielded two initial volumes. The largest class (146 000 particles) showed a very subtle resemblance to the expected protein shape. Two subsequent rounds of 2D classification performed during the refinement and parallel validation stages supported the selection of this volume, as they yielded a higher proportion of 2D averages displaying protein-like features. However, in the parallel 3D branch only one of the three new *ab initio* models generated displayed a faint protein-like morphology. This volume was selected by the consistency check step, which cross-validates the agreement between the 2D and 3D results. At this stage, the workflow had identified a small set of 2D averages containing recognizable protein features, along with a 3D volume that partially resembled the expected structure. Nevertheless, when the selected particles were subjected to non-uniform refinement, the reconstruction did not converge to an interpretable 3D structure. Although the resulting map showed some resemblance to the deposited structure, only very low-resolution features were visible, including a faint lipid belt and poorly defined protein-like densities.

To determine whether the failure was caused by insufficient particle numbers, we tested an alternative strategy. Because the initial 1000 micrographs already yielded approximately 700 000 picked particles, we did not increase the number of micrographs (which accounted for about 26% of the deposited dataset). Instead, we increased the number of particles used during reconstruction, increasing the subset used for *ab initio* volumes generation to 200 000 particles and the 3D classification job to 700 000 particles. Despite this more than threefold increase in particle numbers, the 3D algorithms were still unable to effectively separate empty nanodiscs from nanodiscs containing the protein. These results indicate that the protein signal was too weak to dominate the initial *ab initio* reconstruction and the subsequent 3D classification, preventing the recovery of the target structure.

The original study by Kuzuya *et al.* (2022 ▸) employed a different processing strategy. In their workflow, a 2D classification round with manual class selection was first used to isolate the minority classes containing protein features. This was followed by two rounds of 3D classification prior to final reconstruction, together with contrast transfer function refinement and Bayesian polishing. The final map was obtained after post-processing with masking and was reconstructed using 98 796 particles. Importantly, the authors initially selected 3 005 895 particles, meaning that only 3.2% of these were ultimately used for reconstruction.

Following the strategy described in the original study, we performed two rounds of 2D classification with manual class selection starting from the automated pipeline output of 200 000 particles. These were followed by two rounds of 3D classification with manual selection of the resulting classes and a subsequent non-uniform refinement. This approach produced a reconstruction showing slightly improved structural features of the target protein embedded in the nanodiscs. However, the reconstruction still did not surpass a resolution of 7.7 Å, highlighting the intrinsic difficulty of processing this dataset. Even in the original study, the final reconstruction reached only 4.5 Å.

The failure of EMPIAR-10760 is primarily caused by extreme class imbalance rather than micrograph quality or insufficient particle numbers. This behavior is summarized in Supplementary Figure S13, which provides a step-by-step view of the automated pipeline, highlighting the dominance of empty nanodiscs, the weak protein signal during classification and the inability of both automated and manual strategies to recover a high-resolution structure.

### 3.3. Real-world deployment

The workflow has been successfully implemented and validated as the standard on-the-fly processing solution at the ESRF cryo-EM facility, CM01 (Kandiah *et al.*, 2019 ▸), and the newly commissioned French Collaborative Research Group facility, CM02. The CM01 user program accommodates a wide variety of samples with diverse sizes and structural properties and operates at high throughput, averaging 700–800 images per hour across approximately 100 experiments annually. Each 48 h session may include up to four different samples, increasing the diversity of specimens processed through the pipeline.

Data collection at CM01 uses a Gatan (AMETEK) K3 camera mounted on a Titan Krios microscope. The standard acquisition setting is a nominal magnification of 105 000×, yielding a calibrated pixel size of 0.84 Å. Movies are typically recorded with 40–50 frames per movie (1.6–2.0 s exposure), with average file sizes of about 200 MB (LZW-compressed, gain-unnormalized TIFF). Using two exposures per hole, this setup achieves an average throughput of roughly 750 movies per hour.

Unlike the benchmarking experiments, which were limited to 1000 micrographs, the real-world deployment processes all movies generated during acquisition with no fixed limit. This demonstrates the pipeline’s capability to handle full-scale datasets comprising tens of thousands of movies in multi-day experiments.

#### 3.3.1. Network setup and IT infrastructure for on-the-fly processing

**Figure 9:**
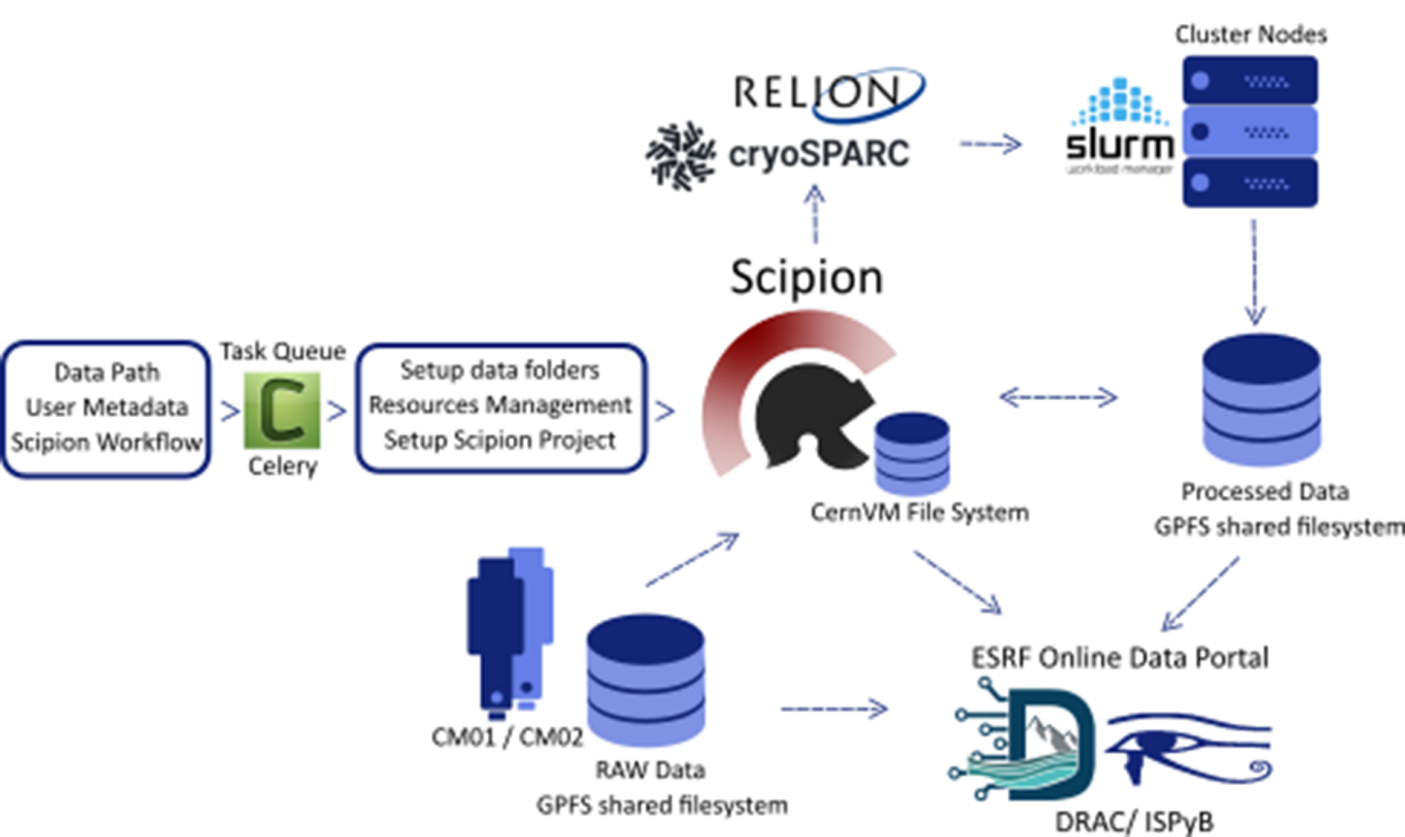
Overview of the *Scipion* on-the-fly workflow deployment at the ESRF. *Scipion* and all associated plugins are installed on the ESRF CernVM File System (CVMFS), while raw and processed data are stored on the GPFS shared filesystem. Users initiate *Scipion* workflows by specifying the data path, relevant metadata and the desired processing template. A *Celery* task is launched to handle the preprocessing steps, including setting up the data directories, allocating computational resources and injecting metadata into the corresponding *Scipion* protocols. *Scipion* subsequently orchestrates downstream protocols (*e.g.CryoSPARC*, *RELION*) that are executed on dedicated *SLURM* cluster partitions. A dedicated *Scipion* protocol automatically transfers acquisition and processing metadata to the *ISPyB*/*DRAC* online platform, enabling real-time monitoring of experiment progress and facilitating user access and data retrieval.

*Scipion*, its plugins and required external software packages are deployed via the ESRF CernVM File System (CVMFS), while raw and processed data are stored on the GPFS shared filesystem. Movies are transferred in real time from the K3 camera computer to the ESRF data center over a 25 Gb fiber-optic network, which enables automatic instantiation of *Scipion* projects at the start of acquisition. This setup ensures efficient, low-latency data transfer and facilitates continuous parallel processing during data collection (Fig. 9 ▸).

The network infrastructure far exceeds the bandwidth requirements for cryo-EM data acquisition. Modern detectors such as the K3 or Falcon 4i generate approximately 1.5–4 GB of data per minute, while a 25 Gbps connection offers a theoretical sustained throughput of about ∼2.5 GB s^−1^ after protocol overhead. This capacity is more than an order of magnitude higher than the detector output, ensuring that real-time data transfer is never a bottleneck for on-the-fly processing.

The storage system uses the GPFS parallel filesystem, designed for high-performance scientific workloads. It supports concurrent read/write operations from multiple compute nodes in the same project directory, avoiding the I/O contention seen with serial filesystems. A memory-backed page pool caches frequently accessed files in RAM, allowing repeated reads to be served directly from memory. At the ESRF, about 10% of system memory is allocated for this purpose, providing roughly 150 GB of high-speed cached data per processing node and significantly accelerating access to intermediate files.

Together, these features ensure the workflow is compute-bound rather than I/O-bound. In practice, operations such as motion correction, particle picking and classification dominate runtime, while data transfer and storage add negligible overhead. Thus, I/O is not a performance bottleneck for the *Scipion* on-the-fly workflow at the ESRF.

#### 3.3.2. Launching the on-the-fly processing

To enable unattended execution, the ESRF data-management system was extended to automatically load and launch workflows at the start of acquisition. Maintained in a version-controlled GitLab repository (ESRF, 2025 ▸), this system uses a template-based strategy, replacing earlier protocol-by-protocol definitions and significantly improving maintainability and flexibility in the following ways.

(i) *Reducing code size and complexity*. Workflow-generation logic was reduced from approximately 1800 lines to fewer than 250 lines of code.
(ii) *Improving modularity*. Pipelines for different use cases (*e.g.* academic versus industrial, tilted versus nontilted samples) are dynamically loaded from templates, eliminating code duplication.
(iii) *Minimizing dependencies*. Templates can be edited, validated and approved without requiring modifications to the central workflow launching system.

Once launched (typically per grid or sample), workflows operate in streaming mode, continuously processing incoming movies. For the cryoEM facility service, integration with *ISPyB* (for SPA) and *ICAT* (for tomography) is achieved by adding specific *Scipion* protocols to the automated pipeline, ensuring that processing results are reported and accessible for real-time monitoring. Users can track data-collection progress and quality via the *ISPyB* interface (Delagenière *et al.*, 2011 ▸). Processed and raw datasets remain accessible via the Data Portal for three months post-acquisition.

All jobs are submitted via the ESRF *SLURM* scheduler, allowing up to three workflows to run concurrently. Resource allocation (CPUs and GPUs) is computed dynamically before launch to optimize cluster utilization. A range of workflow templates is available, from minimal preprocessing (motion correction and CTF estimation) to comprehensive pipelines including 2D classification and preliminary 3D reconstructions. Templates also accommodate tilted datasets, where motion-correction filters, CTF criteria and defocus sampling are modified. Both *CryoSPARC* (the default for academic projects) and *RELION* (used for industrial clients) are supported as processing backends. Templates are stored as JSON files within the *scipion-em-esrf* plugin and version-controlled via GitLab (ESRF, 2025 ▸), ensuring traceability and validation before deployment.

#### 3.3.3. Workflow results

Deployment of the workflow across 34 user datasets at CM01 (Kandiah *et al.*, 2019 ▸) demonstrated robust performance under real experimental conditions. Although each dataset was limited to 200 000 particles due to computing constraints, the outcomes fully met the intended performance goals. Analyses revealed three main reconstruction categories: high quality, where data converged to high-resolution structures; suboptimal, where convergence was achieved but reconstructions exhibited heterogeneity, preferred orientations or other limiting factors that led to lower resolutions; and failed, where no meaningful structure was obtained and only low-resolution, nonspecific density was observed.

Of the 34 experiments analyzed (Table 3 ▸) over 70% converged to interpretable structures, with about half of those achieving high-quality reconstructions at 3–4 Å resolution (particles extracted at 1.5 Å per pixel). The remaining datasets were classified as failed (26.5%). This distribution is consistent with the expected variability in cryo-EM outcomes, which depends heavily on sample quality and preparation. Notably, suboptimal cases were particularly informative, revealing common issues such as heterogeneity or preferred orientation and highlighting the pipeline’s diagnostic value during acquisition.

**Figure 10:**
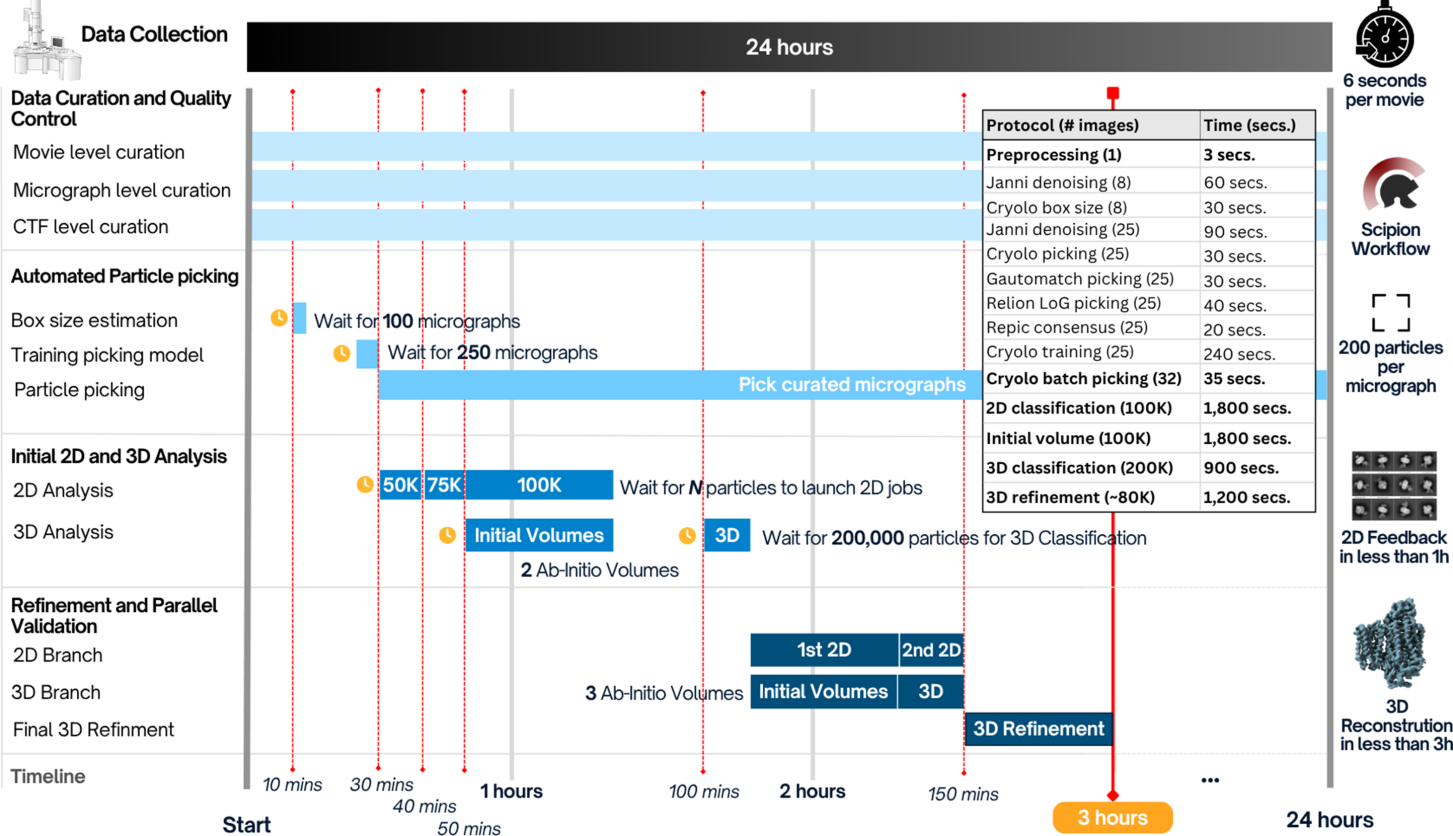
Time diagram of the unattended image-processing pipeline at the ESRF. This figure illustrates the temporal progression of the workflow during live data acquisition. The left panel summarizes the four main stages of the pipeline, while the right panel presents the corresponding execution timeline after acquisition begins. For this example, we assume, based on experimental observations, a microscope acquisition rate of up to 6 s per movie and an average of 200 particles per micrograph. The table on the right reports the execution time of the most relevant protocols; key steps are highlighted in bold and the number of images processed at each step is indicated in parentheses. Preprocessing in this context includes motion correction, data curation and CTF estimation. Prior to particle picking, ‘images’ refer to micrographs; thereafter, they refer to particles. All reported times are approximate and are derived from multiple data acquisition and processing experiments. Not all processing steps are included; the table is intended as a reference to provide an estimate of execution times.

Moreover, Fig. 10 ▸ shows the temporal progression of the workflow during live acquisition. Under typical conditions, the Titan Krios microscope at the ESRF operates at a pace of approximately 6 s per acquired movie. Preprocessing steps (motion correction, CTF estimation and particle picking) in our pipeline were demonstrated to complete in ∼3 s per movie, keeping pace with or exceeding the acquisition speed. Thus, the microscope, not processing, is the limiting factor.

Assuming an average of ∼200 particles per micrograph and a high data-curation acceptance rate, a comprehensive overview of the sample can be achieved within ∼1 h through 2D class averages, while a high-resolution 3D reconstruction can be obtained in under 3 h. Combined with continuous monitoring throughout the data-curation stage from the onset of acquisition, these results demonstrate the pipeline’s ability to provide actionable feedback, enabling real-time informed decision-making.

All performance measurements were obtained on a Ubuntu 24.04 workstation equipped with 64 CPU cores (2× AMD EPYC 9354, 3.25 GHz), 1.48 TB RAM and eight NVIDIA A40 GPUs (Driver Version 550.163.01, CUDA 12.4).

In summary, results from both standardized benchmark and real-world facility deployment demonstrate the broad applicability, high reliability and efficiency of this automated pipeline. On the CryoPPP benchmark datasets, the workflow achieved a 94% processing success rate, with 78% yielding interpretable 3D structures. These findings were validated by deployment at the ESRF CryoEM facility (CM01), where over 70% of user datasets converged to meaningful reconstructions. Beyond success rates, the system’s ability to deliver high-resolution 3D reconstructions in under 3 h, while maintaining preprocessing speeds that exceed data acquisition, establishes it as a critical diagnostic tool for real-time decision-making. By shifting the operational focus from processing to sample quality and microscope throughput, these results lay a robust foundation for automated, on-the-fly analysis in high-throughput cryo-EM facilities.

## 4. Discussion

In this work, we present a fully automated, unattended pipeline for on-the-fly cryo-EM data processing, serving as a robust diagnostic tool for high-throughput facility environments. The pipeline goes beyond simply automating sequential steps; it provides an intelligent workflow that actively assesses data quality, adapts to each specimen and delivers a comprehensive, analysis-ready output. By delivering a full suite of results, including curated micrographs and particles, data-specific picking models, high-quality 2D/3D classes and preliminary 3D maps, the workflow eliminates repetitive early processing steps and allows researchers to immediately focus on high-resolution refinement. The workflow applies to real-time, pre-existing or transferring datasets. Leveraging streaming execution, it processes each new input as it arrives, saving time and providing a reproducible entry point for any reconstruction project, significantly accelerating the path to a final structure.

### 4.1. Benchmarking and operational boundaries

Extensive benchmarking on the diverse CryoPPP dataset demonstrates the pipeline’s robustness and adaptability. With a 94% success rate, 78% of which yielded featured 3D reconstructions, the workflow proved effective across a wide spectrum of challenging targets, from large ribonucleoproteins and membrane proteins to small enzymes and elongated complexes. This performance validates the core design principles of the system: multi-stage quality filters, consensus-based training and integrated parallel 2D/3D validation.

Analysis of ‘suboptimal’ and ‘failed’ cases underscores the pipeline’s diagnostic strengths, as these outcomes typically reflect intrinsic sample limitations, such as preferred orientation, flexibility or contamination, rather than failures of the processing logic. For example, in the ribosome dataset EMPIAR-10526, automated filters rejected a large portion of micrographs due to poor CTF fits and several other quality issues. While manual processing might recover signal from such marginal data through targeted inspection, our pipeline is intentionally conservative; it prioritizes rapid feedback and high-quality data curation over the recovery of borderline images. Notably, some heavily contaminated micrographs in this dataset passed CTF fit criteria, reinforcing the observation that resolution metrics alone are insufficient and that a combination of multiple, complementary quality-control strategies is needed to ensure robust data curation. In a real-world scenario, such rapid feedback is invaluable, allowing users to recognize problematic acquisitions early and adjust or terminate experiments, ultimately saving days of microscope time.

Specific challenges were observed with membrane proteins, such as the pannexin-1 nanodisc dataset (EMPIAR-10760). Here, a severe class imbalance caused by empty nanodiscs, where only 3.2% of picked particles contained the target protein (Kuzuya *et al.*, 2022 ▸), complicated automated 3D processing. In contrast, EMPIAR-10737, which the workflow successfully reconstructed, contained a much higher proportion of protein-occupied nanodiscs, allowing consistent particle identification. These examples highlight the biochemical and sample-preparation challenges of membrane proteins, where empty nanodiscs can dominate and overwhelm automated analysis.

Similarly, the workflow’s operational boundaries are defined by pronounced conformational flexibility (*e.g.* EMPIAR-10590) or exceptionally low signal-to-noise ratios (*e.g.* EMPIAR-11057). While highly heterogeneous systems such as the 70S ribosome (EMPIAR-10406) can generally be handled by separating distinct states during 3D classification, pronounced flexibility (*e.g.* the human BAF complex, EMPIAR-10590) inherently limits the achievable resolution. Further improvement requires advanced heterogeneity analysis or manual refinement beyond the scope of an automated workflow.

Finally, while denoising strategies can mitigate low contrast, intrinsic data constraints such as strong preferred orientation (EMPIAR-10387) or highly elongated complexes (EMPIAR-10669) still occasionally require expert intervention. Preferred orientation or limited particle numbers reduce angular coverage and reconstruction quality; although the workflow flags these issues early, the data constraints remain and should prompt users to adjust sample-preparation or imaging strategies. Furthermore, highly elongated complexes can challenge automated parameter estimation, leading to suboptimal box sizes and incomplete reconstructions that necessitate manual adjustment.

### 4.2. Real-world deployment and performance

Deployment at the ESRF CM01 facility confirmed the pipeline’s robustness under real-world conditions. Approximately 70% of 34 user datasets converged to interpretable structures, with nearly half achieving high-resolution reconstructions (3–4 Å). This slightly lower success rate compared with the CryoPPP benchmark is expected; facility experiments occasionally involve novel or unstable samples whose biochemical or biophysical properties remain uncertain, unlike EMPIAR datasets, which represent well characterized and already successful projects. In this context, the pipeline’s ability to rapidly distinguish promising from unproductive samples and generate preliminary structures during acquisition is invaluable, enabling researchers to confirm that their target protein has been successfully captured while also detecting potential issues such as heterogeneity, dissociation or preferred orientation within hours.

Another key strength is the workflow’s real-time performance. At the ESRF CM01 facility (Titan Krios microscope, Gatan K3 detector, 750 movies per hour), preprocessing outpaces data acquisition, ensuring that analysis never lags behind. The ability to generate a preliminary 3D reconstruction in under 3 h, often before the data-collection session has even finished, fundamentally changes the experimental paradigm. It allows operators to move from passive data collection to active, informed decision-making, such as implementing tilted data collection (Wagner *et al.*, 2019 ▸) when preferred orientation is detected.

### 4.3. Challenges in comparative validation

A significant challenge in the objective validation of automated reconstructions lies in the lack of standardization across deposited structural data. While rigid-body fitting or quantitative similarity metrics could, in principle, provide a numerical basis for comparison, EMDB maps often undergo undocumented post-processing (*e.g.B*-factor sharpening or masking) that is difficult to replicate without the original metadata. Furthermore, discrepancies in particle-extraction parameters, such as box size and scaling, further hinder the use of automated similarity scoring. A fully standardized and unbiased comparison would require access to original particle coordinates and the application of consistent particle-extraction protocols and identical reconstruction procedures to complete datasets; conditions that are currently not met for the majority of publicly available data. Moreover, performing such a high-intensity re-analysis across all benchmarked datasets would require substantial computational resources, making it impractical within the scope of this study.

Given these constraints, we adopted an evaluation framework focused on global structural agreement and interpretability. This approach ensures a fair assessment of the pipeline’s primary goal: providing rapid, actionable feedback on the overall 3D structure of the target macromolecule, rather than attempting to replicate the final, manually tuned results of a full-scale structural study.

Notably, despite these hurdles, several reconstructions generated by the automated pipeline reached resolutions comparable to those of the deposited structures. These results must be interpreted in light of the methodological differences: while published studies typically involve extensive manual optimization and symmetry imposition, our workflow applies a fully standardized approach, using fixed extraction parameters and no imposed symmetry, to minimize model bias and ensure general applicability. Furthermore, datasets processed within our workflow were capped at 1000 micrographs and 200 000 particles, which is often substantially fewer than the counts used in the original published studies. Since reconstruction resolution typically improves with increased particle numbers, the fact that several datasets yield comparable resolutions highlights the efficiency of the workflow in extracting meaningful structural information from reduced data. To ensure consistency, resolution was assessed using the same gold-standard Fourier shell correlation (FSC) 0.143 criterion used in the original studies.

### 4.4. Future outlook and framework integration

While the pipeline represents a major advance, several avenues for improvement remain. Challenges persist with elongated particles, where box-size estimation can be sub­optimal, and with contaminant-dominated datasets, where classification occasionally struggles to isolate minority classes. Future enhancements will focus on the dynamic adjustment of box sizes for nonglobular particles and specialized filtering steps to better separate target proteins from common contaminants, such as empty nanodiscs or micelles. Furthermore, while the current workflow relies on the analysis of a fixed particle set, a transition to a fully dynamic, streaming-based model, in which 2D and 3D analyses are continuously updated as new data arrive, would provide even greater real-time feedback.

Currently, the pipeline does not implement the automatic termination of data acquisition. Quality-control protocols are designed to monitor and flag potential issues, but decisions regarding the continuation or suspension of a session remain under user control. This design choice reflects the inherently context-dependent nature of acquisition decisions, which benefit from expert supervision and experimental awareness beyond what can be inferred from image-processing metrics alone. However, in critical failure scenarios, such as sustained acquisition instability or persistently high rejection rates, the workflow could, in principle, be extended to automatically halt execution and notify the operator. A further step would involve direct integration with microscope control software to guide acquisition. This would complete the anticipated ‘feed-forward’ loop, where on-the-fly processing directs the microscope to the optimal grid regions and acquisition conditions, thereby minimizing computational expenditure and maximizing instrument time.

Similarly, the pipeline does not yet perform automatic dynamic parameter tuning in response to fluctuating data quality. Instead, robustness is currently achieved through conservative, yet intentionally nonrestrictive, default thresholds, empirically validated across diverse datasets and acquisition strategies. These thresholds act as quality-gating mechanisms that prevent severely degraded data from propagating downstream while avoiding overly aggressive filtering that could discard valuable information. The implementation of adaptive parameter adjustments based on real-time quality metrics represents a natural extension of this framework and remains a key objective for future development.

Implementing this pipeline within the open-source *Scipion* framework provides a robust foundation for these future enhancements. *Scipion*’s modular design enables the seamless integration of new algorithms and tools, allowing the workflow to adapt to emerging challenges without the restrictions of closed, all-in-one processing suites. Its open-source nature ensures transparency and reproducibility, as every processing step remains inspectable, validated and shareable, qualities that benefit the broader scientific community. Furthermore, the framework enforces traceability by automatically recording parameter choices and processing decisions, which is critical for scientific rigor and data-sharing compliance. These features make *Scipion* an ideal platform for both current deployment and continuous evolution. As cryo-EM facilities continue to increase throughput, automated diagnostic workflows will become an essential component of data-collection strategies, enabling microscopes to operate not only as imaging instruments but also as real-time structural discovery platforms.

## 5. Conclusion

We have developed and validated a fully automated, on-the-fly processing pipeline that addresses key bottlenecks in the cryo-EM workflow. Its main strength is acting as an intelligent diagnostic tool delivering rapid, reliable data-quality assessment rather than merely pursuing ultimate resolution. Extensive benchmarking and real-world deployment demonstrate its robustness across diverse samples, as well as its speed, which matches or exceeds the pace of modern data acquisition. By providing comprehensive set of results before data collection ends, this method empowers researchers to make critical decisions in real time, maximizing microscope efficiency. The pipeline requires no user scripting; workflows launch directly from *Scipion* using ready-to-use JSON templates publicly available in the WorkflowHub collection ‘CryoEM Facility Workflows’ (Scipion CNB, 2025 ▸). This work is a significant step towards making high-throughput cryo-EM more efficient, accessible and intelligent.

## Supplementary Material

Software specifications and model weights, Supplementary Figures and image-processing table for the complete CryoPPP dataset. DOI: 10.1107/S205979832600656X/bar5004sup1.pdf

Supplementary Table S3. DOI: 10.1107/S205979832600656X/bar5004sup2.xlsx

## Figures and Tables

**Table 1:** General overview of CryoPPP benchmarking results

3D final reconstruction	Total percentage of 32 entries (%)	FSC range resolution (Å)
High quality	78 (25/32)	3.1–6.7
Suboptimal	16 (5/32)	3.7–11.9
Failed	6 (2/32)	N/A

**Table 2:** Image-processing summary ‘3D map result assessment’ indicates whether the resulting map displayed recognizable, protein-like features consistent with the target macromolecule. Blue highlights mark key aspects of the image processing that may have influenced the final 3D structure. ‘Used mics.’ denotes the percentage of the total EMPIAR dataset processed (limit: 1000 micrographs per entry). ‘Ref. part.’ indicates the particle count used in the originally deposited EMDB reconstruction. ‘Acc. curation’ represents the percentage of micrographs retained after quality filtering. ‘Final/initial particles’ shows the number of particles used in the final refinement compared with those initially selected from the curated set of micrographs, capped at 200 000 particles.

EMPIAR	Protein type	Molecular weight (kDa)	Used mics. (%)	Deposited/FSC resolution (Å)	Ref. part.	3D map result assessment	Acc. curation (%)	Particle diameter/estimate (Å)	Final/initial particles	Features of micrographs
10406	Ribosome (70S)	633	36.8	2.7/3.1	52000	High quality	58.8	240/232	23000/74000	Monodispersed particles; moderate edge texture
10576	Nuclear protein (DNA)	290	50.4	2.9/3.2	194000	High quality	97.4	180/208	103000/200000	Low contrast; hard to recognize and to pick
10737	Membrane protein (*E. coli*)	156	29.0	2.2/4.1	95000	High quality	75.2	179/172	56000/177000	Monodispersed particles; sufficient contrast
10184	Aldolase	150	62.0	2.4/3.2	187000	High quality	47.5	100/90	111000/200000	Monodispersed compact particles
11057	Hydrolase	149	11.9	2.8/3.1	44000	High quality	80.4	140/118	88000/161000	Difficult to identify; suboptimal concentraction; ice issues
10389	Metal-binding protein	1042	23.1	2.0/4.0	98000	Suboptimal	65.4	200/165	6000/30000	Abundant ice patches; low part/mic
10671	Signaling protein	77	17.0	3.5/6.1	284000	Suboptimal	96.6	110/86	90000/200000	Extremely small; high density of particle
10669	Proteasome (plant)	1682	2.2	3.2/5.9	289000	Suboptimal	95.3	500/229	27000/101000	Carbon edges; dispersed and distinct particle views
10387	Viral protein (DNA)	186	49.5	2.8/3.7	139000	Suboptimal	84.3	168/200	21000/40000	Highly aggregated protein; difficult to pick
10590	BAF complex (human)	1000	20.6	7.8/11.9	89000	Suboptimal	94.0	236/250	29000/181000	High contrast; monodisperse; ice contaminations
10526	Ribosome (50S)	1086	90.7	2.8/NA	21000	Failed	1.9	400/NA	NA	Extreme ice contamination; varying thickness
10760	Membrane protein	322	26.0	4.5/8.3	99000	Failed	99.6	130/122	66000/200000	Abundant ice patches; monodisperse; sufficient contrast

**Table 3:** General overview of ESRF real-world results

3D final reconstruction	Total percentage of 34 entries (%)	FSC range resolution (Å)
High quality	35.3 (12/34)	3–4
Suboptimal	38.2 (13/34)	4–8
Failed	26.5 (9/34)	N/A

## Data Availability

This study analyzes both publicly available cryo-EM data from EMPIAR and private user data collected at the ESRF, which cannot yet be shared or disclosed. The accession numbers for the public datasets correspond to those included in the CryoPPP collection (Dhakal *et al.*, 2023 ▸), all of which are cited within the paper. The workflow templates used in this study are publicly available through the WorkflowHub collection ‘CryoEM Facility Workflows’ (Scipion CNB, 2025 ▸), which also provides detailed documentation. This collection includes (i) a manual for using *Scipion* in the context of a cryo-EM facility, covering on-the-fly processing, workflow and template design, and integration with queue systems, and (ii) a detailed guide describing the available templates, image-processing steps, implementation aspects and required software. The collection can be accessed at https://workflowhub.eu/collections/31. All software used in this work is freely available through the official *Scipion* GitHub organization, which hosts the corresponding plugins: https://github.com/scipion-em. Any additional information required to reproduce or reanalyze the results presented in this paper is available from the lead contact upon request.
