# Supplementary material for: An unattended image-processing pipeline for on-the-fly quality assessment and 3D exploration in cryo-EM
Source: Acta Crystallogr D Struct Biol. 2026 Jul 28;82(Pt 8):915–39. doi: 10.1107/S205979832600656X (PMC13431645; doi:10.1107/S205979832600656X)
Supplement: Supplementary file 1 [file d-82-00915-sup1.pdf]

STRUCTURAL
BIOLOGY

Volume 82 (2026)

Supporting information for article:

An unattended image-processing pipeline for on-the-fly quality assessment and 3D exploration in cryo-EM

Daniel Marchán Torres, Pablo Conesa, Alberto Garcia, Mikel Iceta, Ludovic Broche, Marcos Gragera, Romain Linares, Hok Sau Kwong, Javier Chichón, Olof Svensson, Rocío Arranz, Grégory Effantin, Eaazhisai Kandiah, José María Carazo and Carlos O. S. Sorzano

S1 Software Specifications and Model Weights

To ensure the reproducibility of the automated pipeline, this appendix details the software versions and deep-learning model weights employed. All processing was performed within the Scipion (v3.8.1 - Eugenius) framework.

Table S1: Software Versions and Pipeline Tasks. Summary of the software packages, their versions, and their specific roles within the automated workflow.

Software / Plugin	Version	Task
Scipion	v3.8.1 - Eugenius	Workflow Framework
Xmipp	v3.25.06 - Rhea	Image Processing Suite
Relion	v5.0	Particle picking, 2D and 3D processing
Cryosparc	v4.6.0	2D Classification and 3D Processing
Ctffind5	v5.0.2	CTF Estimation
MotionCor3	v1.1.2	Motion Correction
Gautomatch	v0.56	Particle Picking
Sphire	v1.9.9	Denoising and Particle Picking
Repic	v1.0.0	Ensemble Picking
EM-facilities	v3.2.0	Processing Tools
Cryoassess	v1.0.0	Quality Assessment (Mics and 2D)
Miffi	v1.0.0	Quality Assessment (Mics)

Table S2: Pre-trained Deep Learning Model Weights. Specific model weights used for automated denoising, particle picking, and class assessment.

Algorithm / Tool	Model Weights / File Name	Task
2D-Assess	2dassess_062119.h5	2D Class Selection
crYOLO	gmodel_phosnet_202005_N63_c17.h5	Particle Picking
JANNI	gmodel_janni_20190703.h5	Micrograph Denoising
Miffi (Mic Filter)	miffi_v1.pt	Micrograph Quality Filtering

S2 Supplementary Figures for the Pipeline Methodology

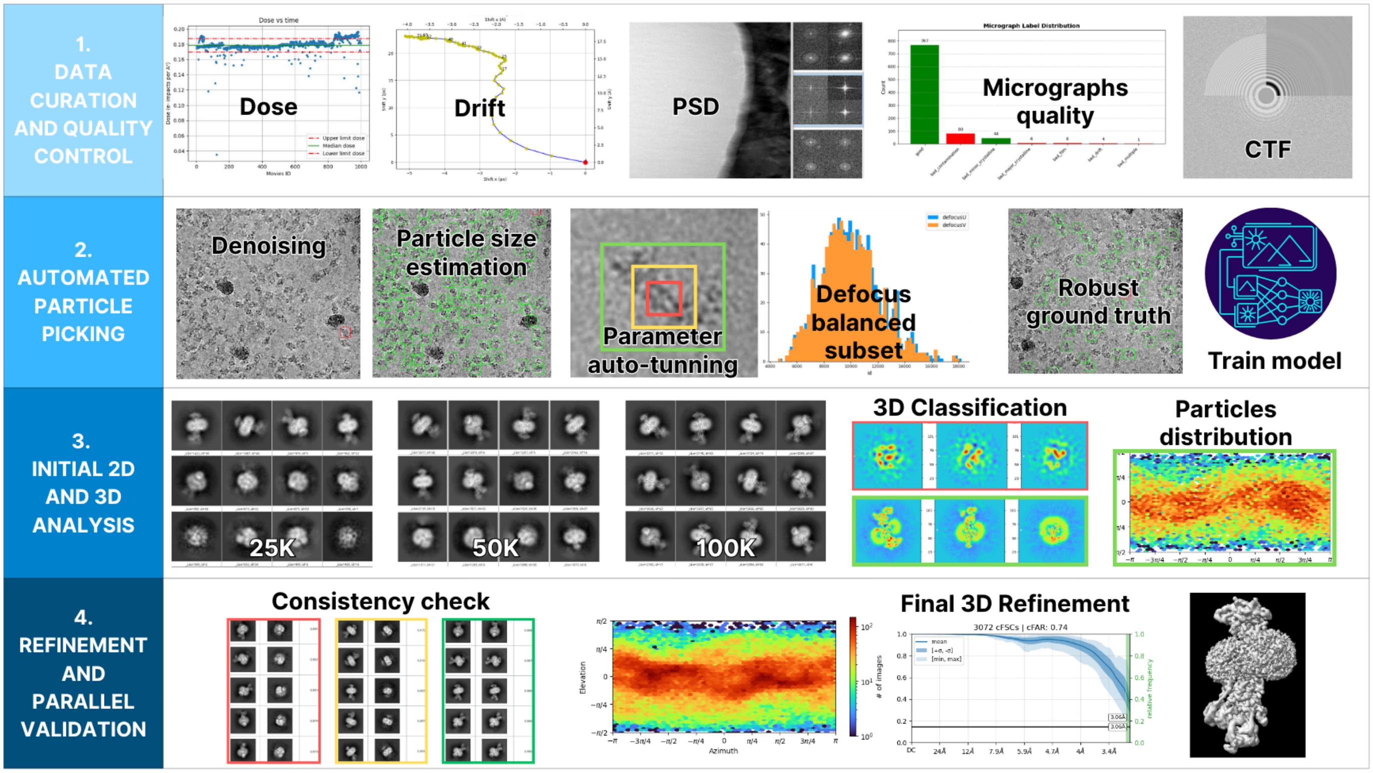

Figure S1: Detailed graphical summary of the four main stages of the automated image processing pipeline.

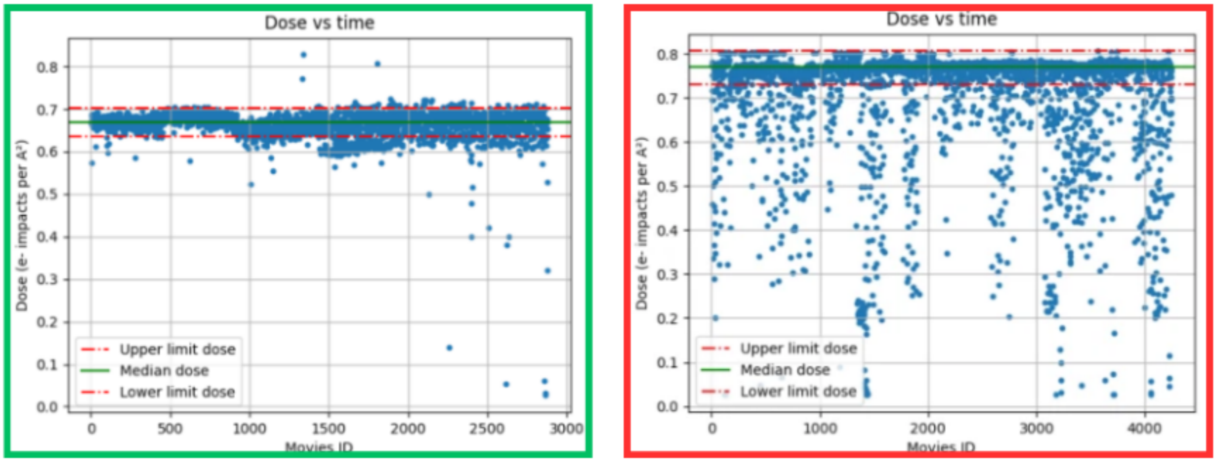

Figure S2: Examples of Dose Analysis Plots. Highlighted in green is an optimal acquisition exam-ple showing a stable and consistent electron dose. Highlighted in red is an unstable acquisition, displaying clear dose inconsistencies during data collection. Such erratic behaviour can indicate issues with the electron source or variations in ice thickness. The global median (μ) is shown as a green line, while user-defined d ifference t hresholds a re s hown i n r ed, m arking t he m ovies t hat do not meet the filtering criteria.

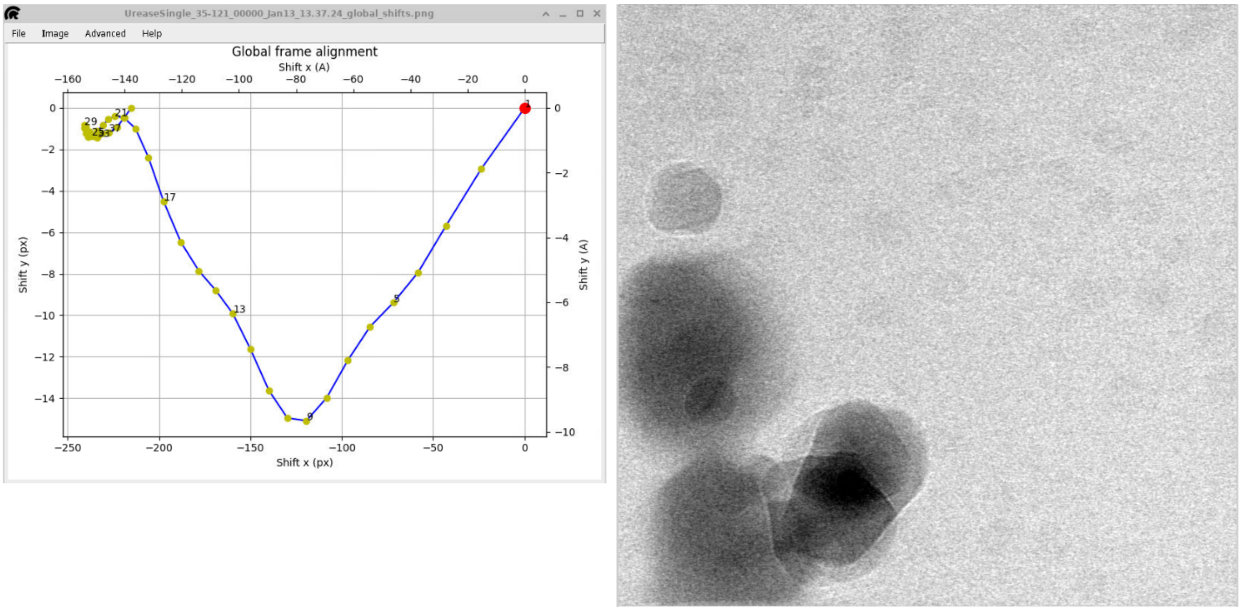

Figure S3: Example of a micrograph discarded by the Max Shift Filter. On the left, the trajectory plot shows the global alignment path in both the X and Y directions. Each yellow dot represents an individual frame, while the blue line indicates the movement between consecutive frames. On the right, the corresponding faulty micrograph is shown, exhibiting a large maximum drift per frame (15 Å) and a substantial overall global drift (154 Å). Micrograph is from EMPIAR- 10389.

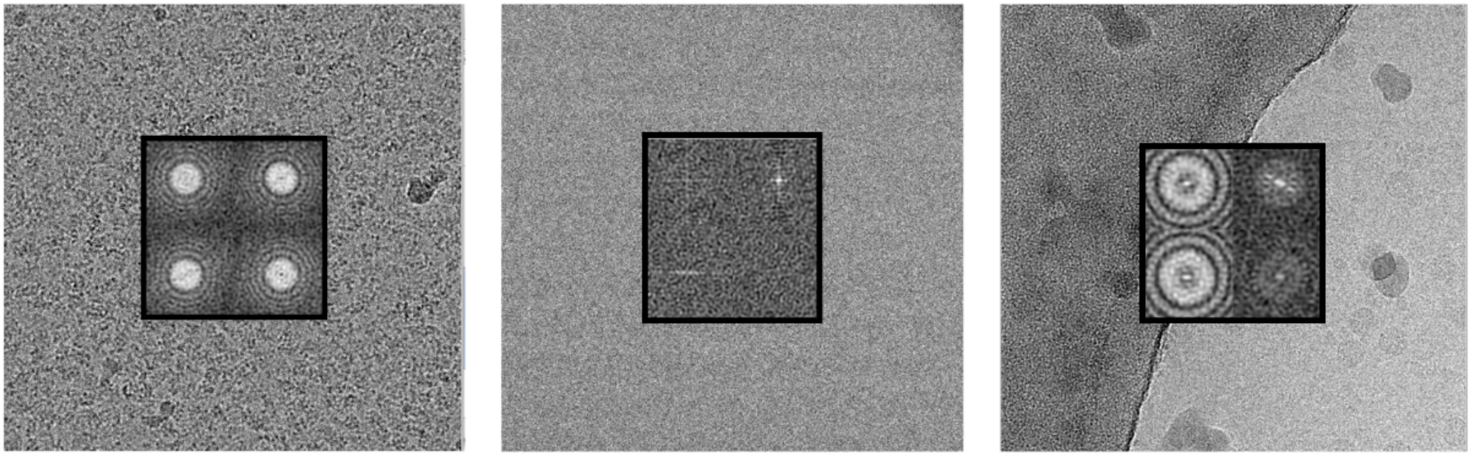

Figure S4: Examples of PSD Analysis Plots. The left micrograph shows an ideal image with a uniform signal across the field (high c orrelation: 0 .8; l ow s tandard d eviation: 0 .02). T he center micrograph shows a low-contrast, empty image (low correlation: 0.1; low standard deviation: 0.03). The right micrograph illustrates a two-texture case acquired at the edge of a hole, with half of the image over the foil (medium correlation: 0.56; high standard deviation: 0.2). Micrograph are from EMPIAR-11051.

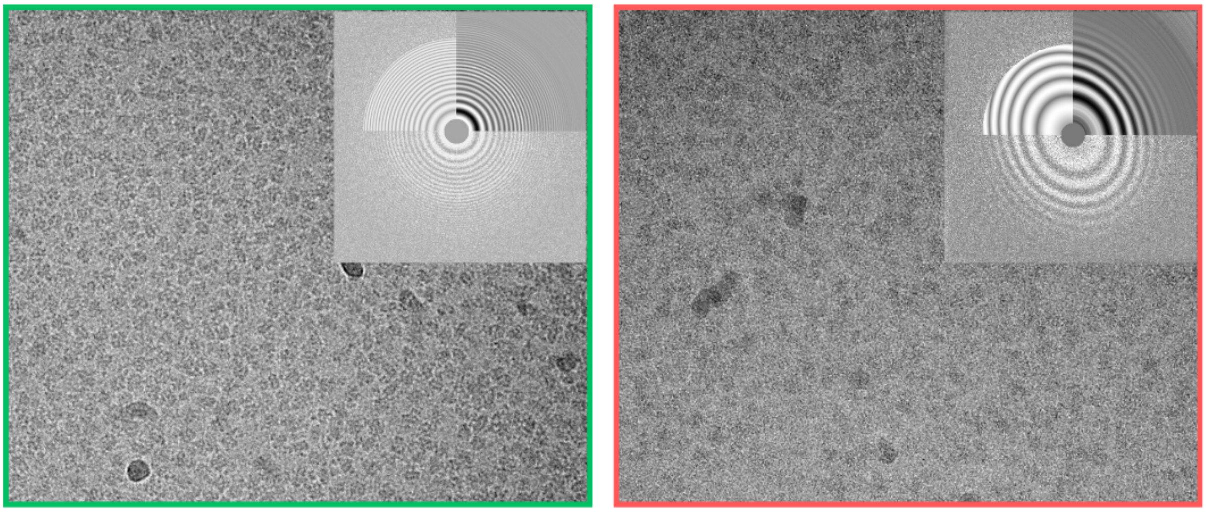

Figure S5: Examples of CTF estimation and astigmatism. Highlighted in green is an ideal CTF with an astigmatism ratio of 0.004, and in red an astigmatic micrograph with an astigmatism ratio of 0.32, displaying the characteristic oval Thon ring pattern typical of astigmatic micrographs. Micrographs are from EMPIAR-11057.

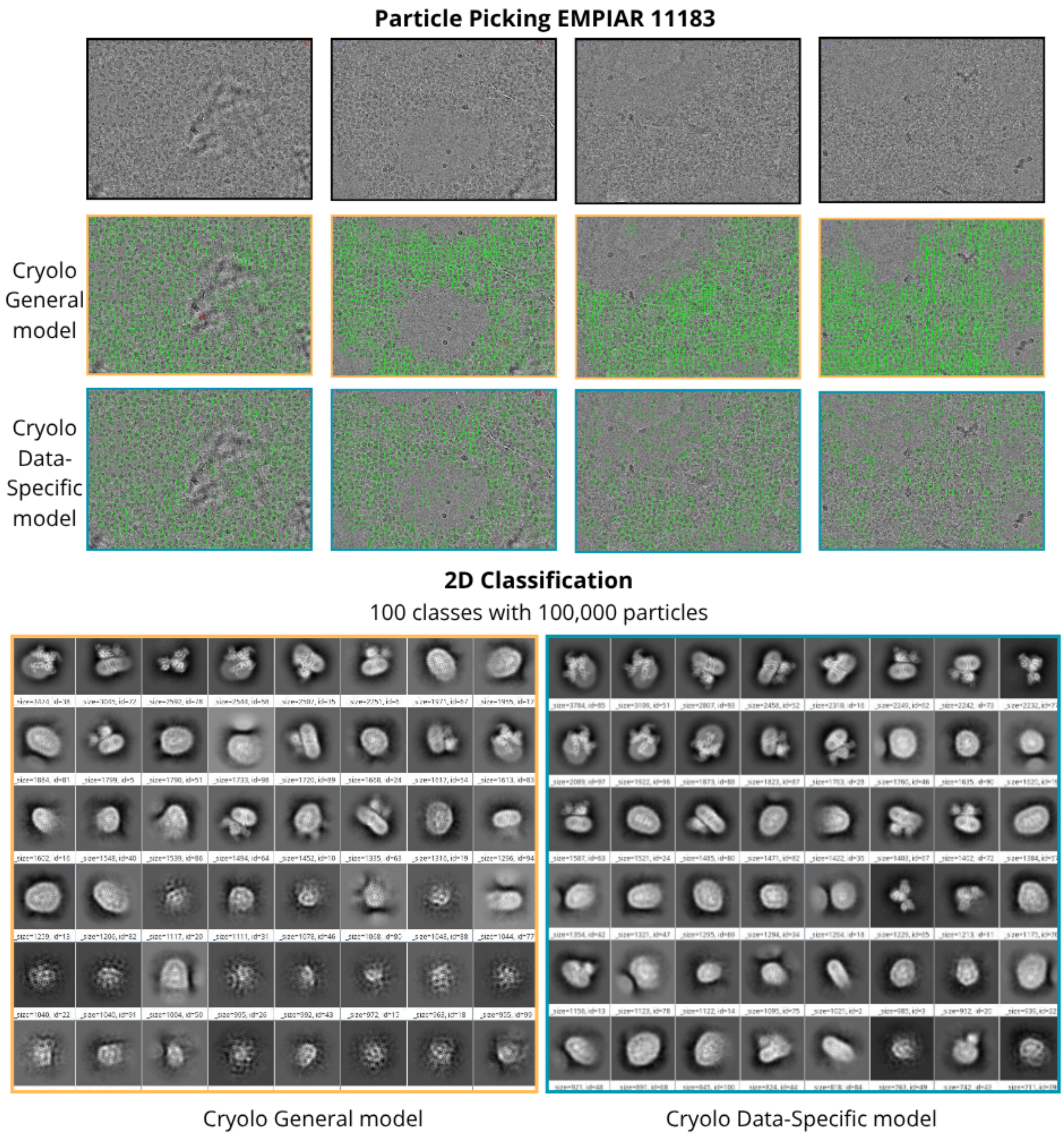

Figure S6: Comparison of particle picking performance between the general and data-specific models. The figure is organized into two panels. (**Top**) Visual comparison of particle picking results using the crYOLO general model (orange) and the data-specific model (blue) trained within the automated pipeline. The general model selects more particles, including regions with aggregation and potential contaminants, while the data-specific model demonstrates improved selectivity and better discrimination of true particles. (**Bottom**) Quantitative evaluation of both picking strategies. Each model was applied to the same set of micrographs, and the first 100,000 particles were used for downstream processing. A 2D classification into 100 classes was performed using CryoSPARC. The data-specific model produced higher-quality class averages, with more well-defined classes, a greater variety of views, and sharper structural features. This indicates improved precision, as better results are achieved using the same number of particles. Data from EMPIAR-11183.

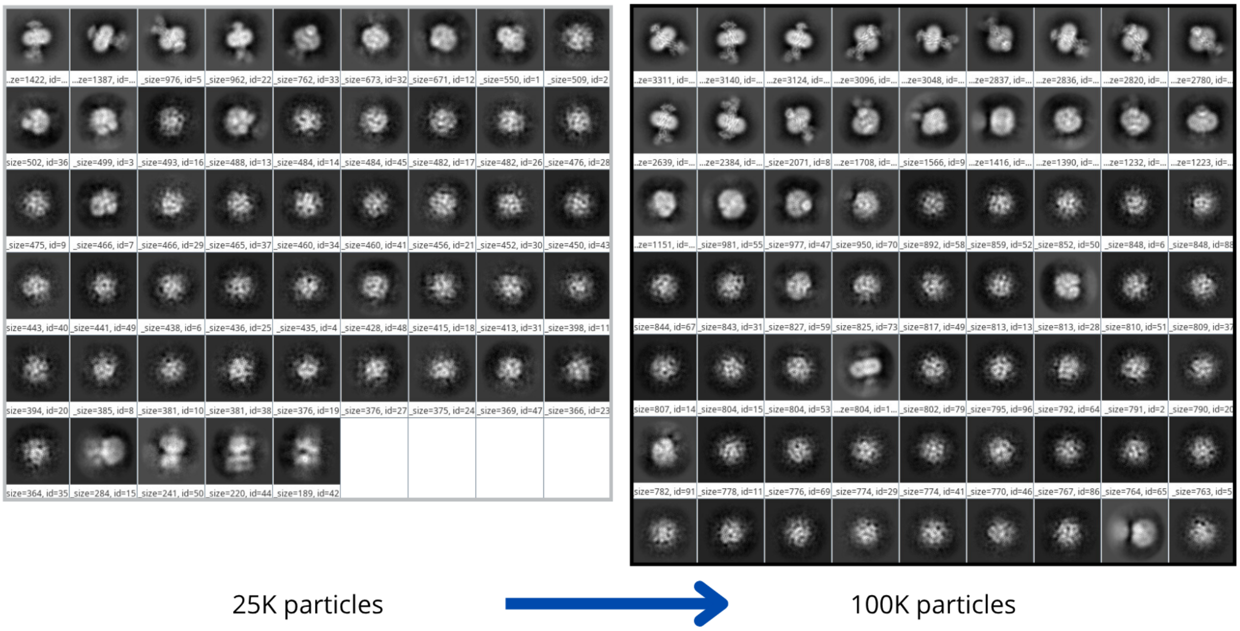

Figure S7: Example of 2D class average evolution with cumulative batches. On the left, a 2D classification of 50 classes with 25,000 particles; on the right, a 2D classification of 100 classes with 100,000 particles. As more particles become available, the 2D averages show greater detail, and new particle views begin to appear. Data from EMPIAR-11057.

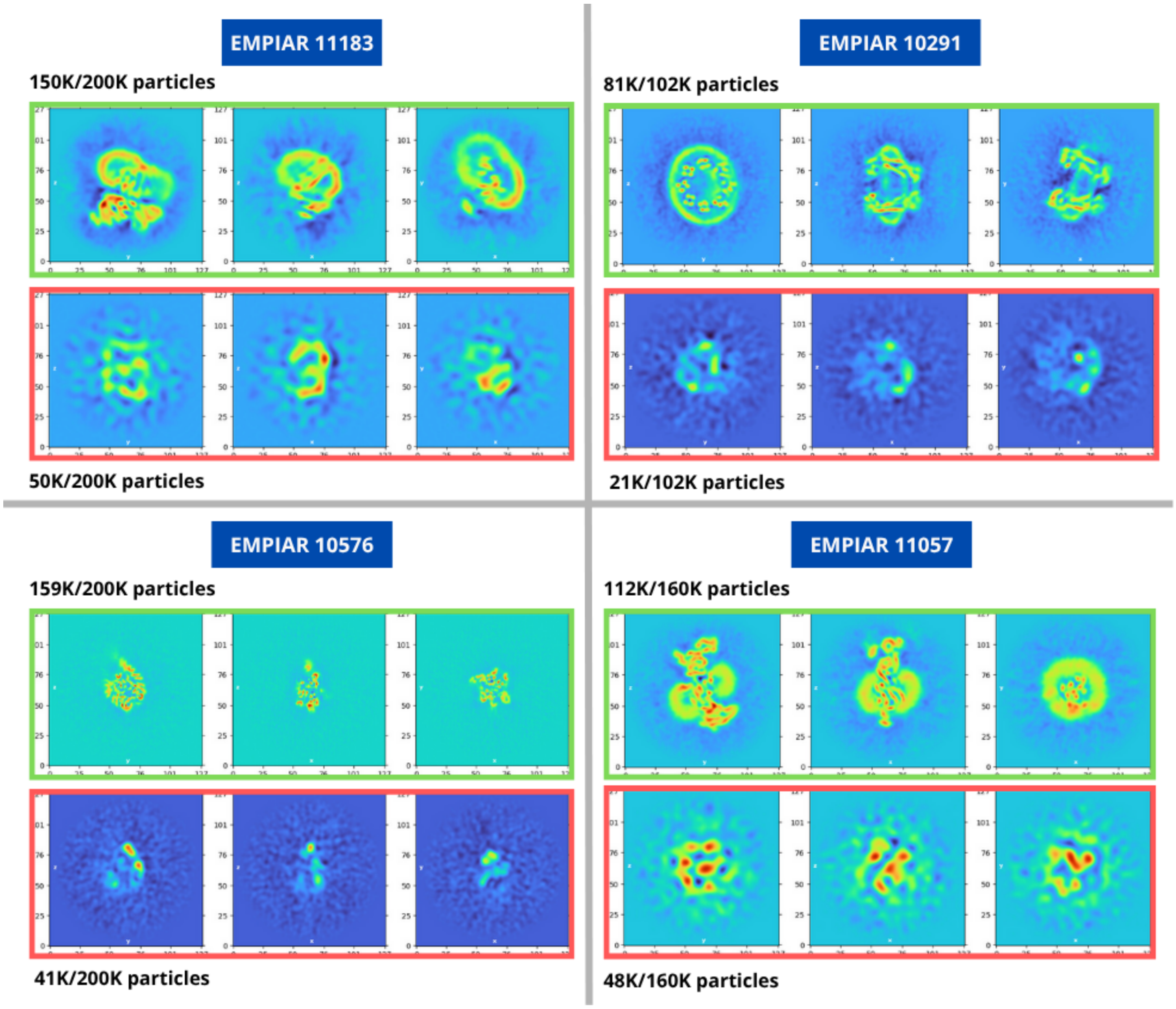

Figure S8: 3D Classification examples. Each quadrant corresponds to an EMPIAR entry used to illustrate particle separation during 3D classification with refinement between two *ab initio* models (using CryoSPARC algorithms within Scipion). The class with the larger particle population is highlighted in green, while the smaller class is highlighted in red. Particles belonging to the smaller 3D class are excluded from further processing. Data are from EMPIAR-11183, EMPIAR-10291, EMPIAR-10576, and EMPIAR-11057.

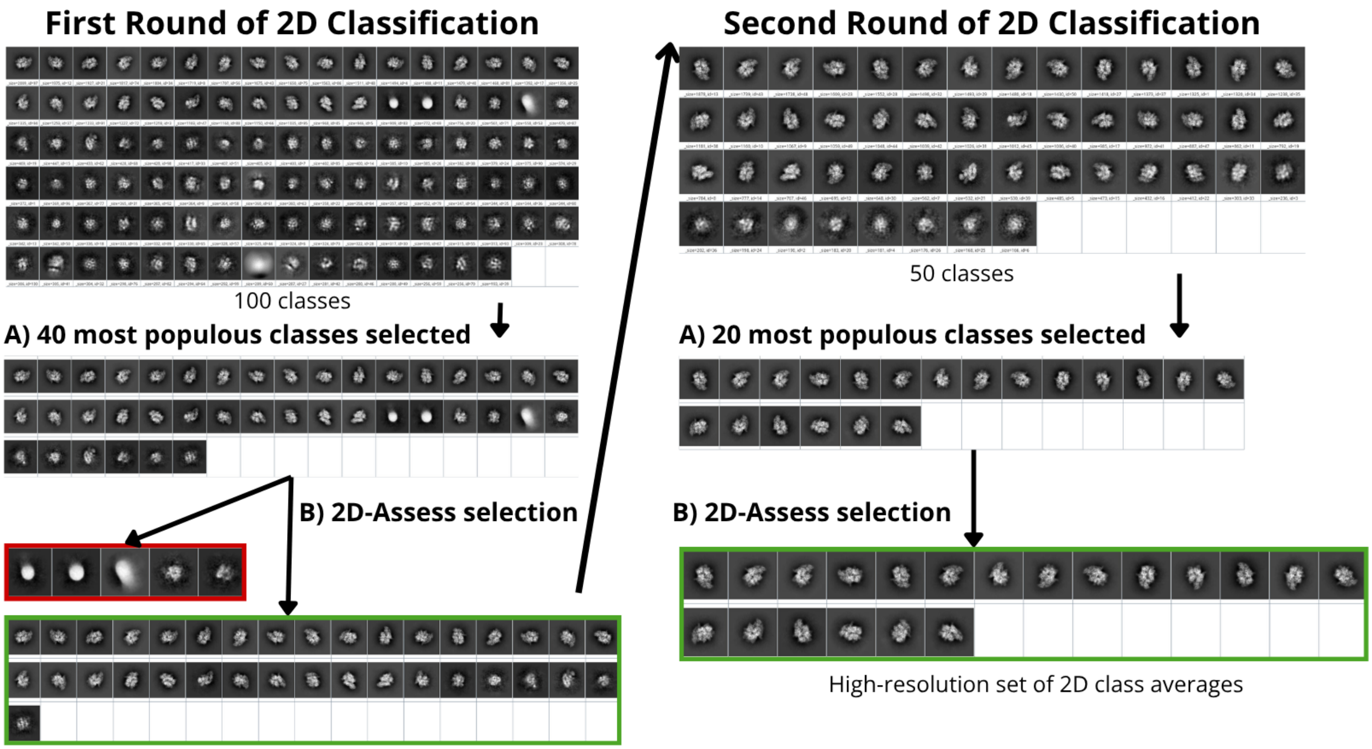

Figure S9: 2D Branch: Consecutive classification and selection strategy. The figure illustrates the two-round workflow that combines 2D classification with automated selection criteria. (**Left**) First round of 2D classification, followed by two selection steps: (A) selection of the 40 most populous classes, and (B) evaluation using 2D-Assess. Classes rejected by the deep learning model are shown in red, while accepted classes are in green. Particles from accepted classes are retained for the second round. (**Right**) Second round of 2D classification using the curated particles, followed by the same selection criteria: (A) selection of the 20 most populous classes, and (B) 2D-Assess evaluation. In this example, no classes were rejected in the final 2D-Assess step. This two-stage process yields a refined set of high-quality, high-resolution 2D class averages. All 2D classifications were performed using CryoSPARC. Data from EMPIAR-11051.

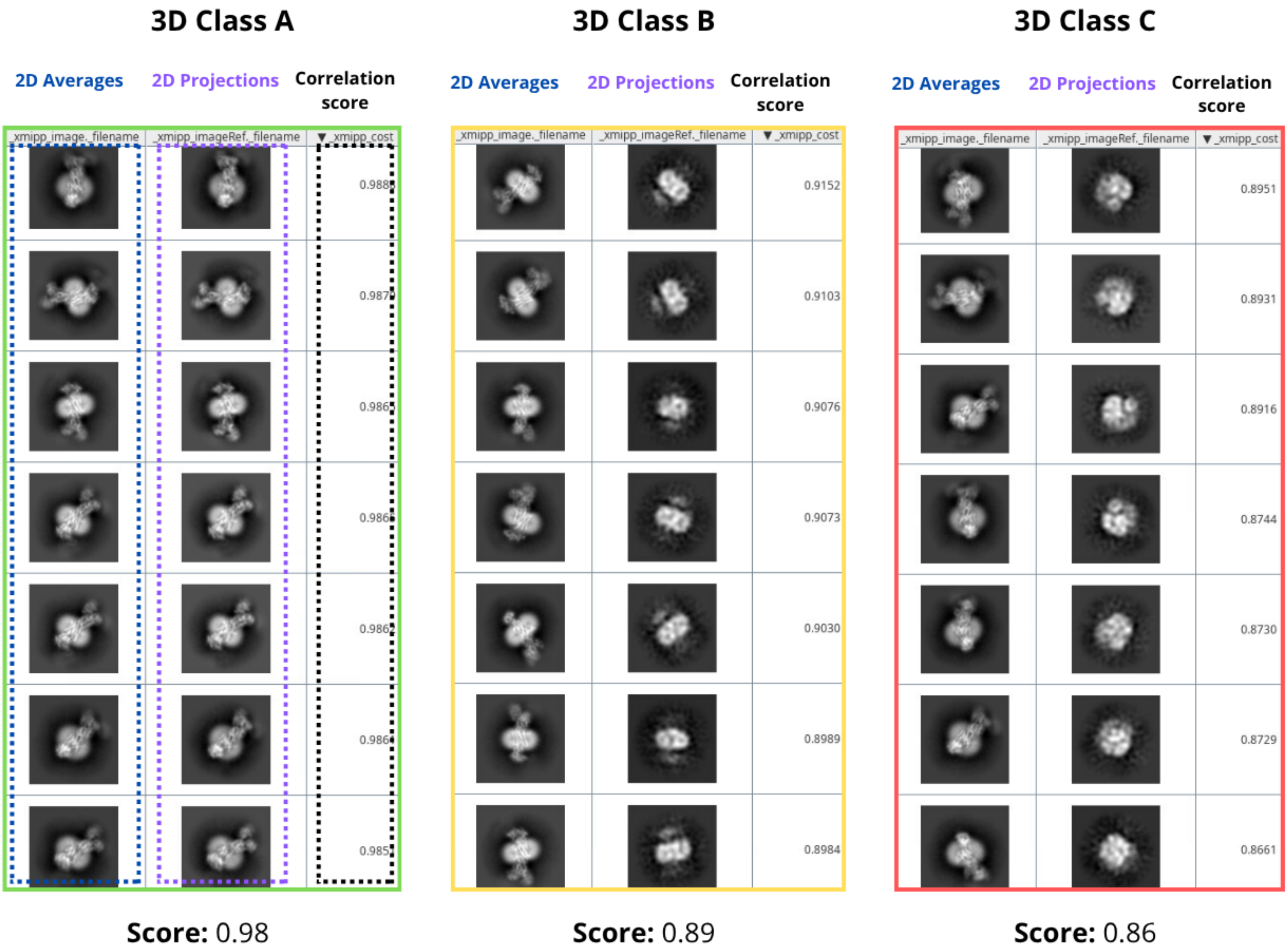

Figure S10: Cross-Validation of 2D and 3D Results. The titles at the top indicate the 3D class being evaluated. The blue column shows the high-quality 2D averages from the 2D branch, and the adjacent purple column shows the 2D projections of the corresponding 3D-class volume. These projections represent the best matches to the 2D averages after an exhaustive search. The Score below refers to the average correlation between the set of 2D averages and their best-matching 2D projections. The 3D class with the highest score is selected to continue in the image-processing pipeline.Data is from EMPIAR-11057.

S3 Image processing table for the complete CryoPPP dataset

Table S3 (provided as a separate spreadsheet) summarizes the image processing results for the complete CryoPPP dataset. Unlike (Table 2), which presents only a few representative high-quality cases (5 out of 25), this table includes the full set of 32 CryoPPP EMPIAR entries. “3D Map Result Assessment” indicates whether the resulting map displayed recognizable, protein-like features consistent with the target macromolecule. “Used mics” refers to the percentage of total micrographs

deposited in EMPIAR that were included in processing, with a maximum of 1,000 per entry. “Accepted data curation” indicates the percentage of micrographs that passed the quality filters from the initial processing set. “Final Particles” shows the number of particles retained in the final refinement relative to those initially selected (“Initial Particles”) from the curated micrographs, capped at 200,000 particles.

S4 Supplementary Figures for the Pipeline Benchmarking Results

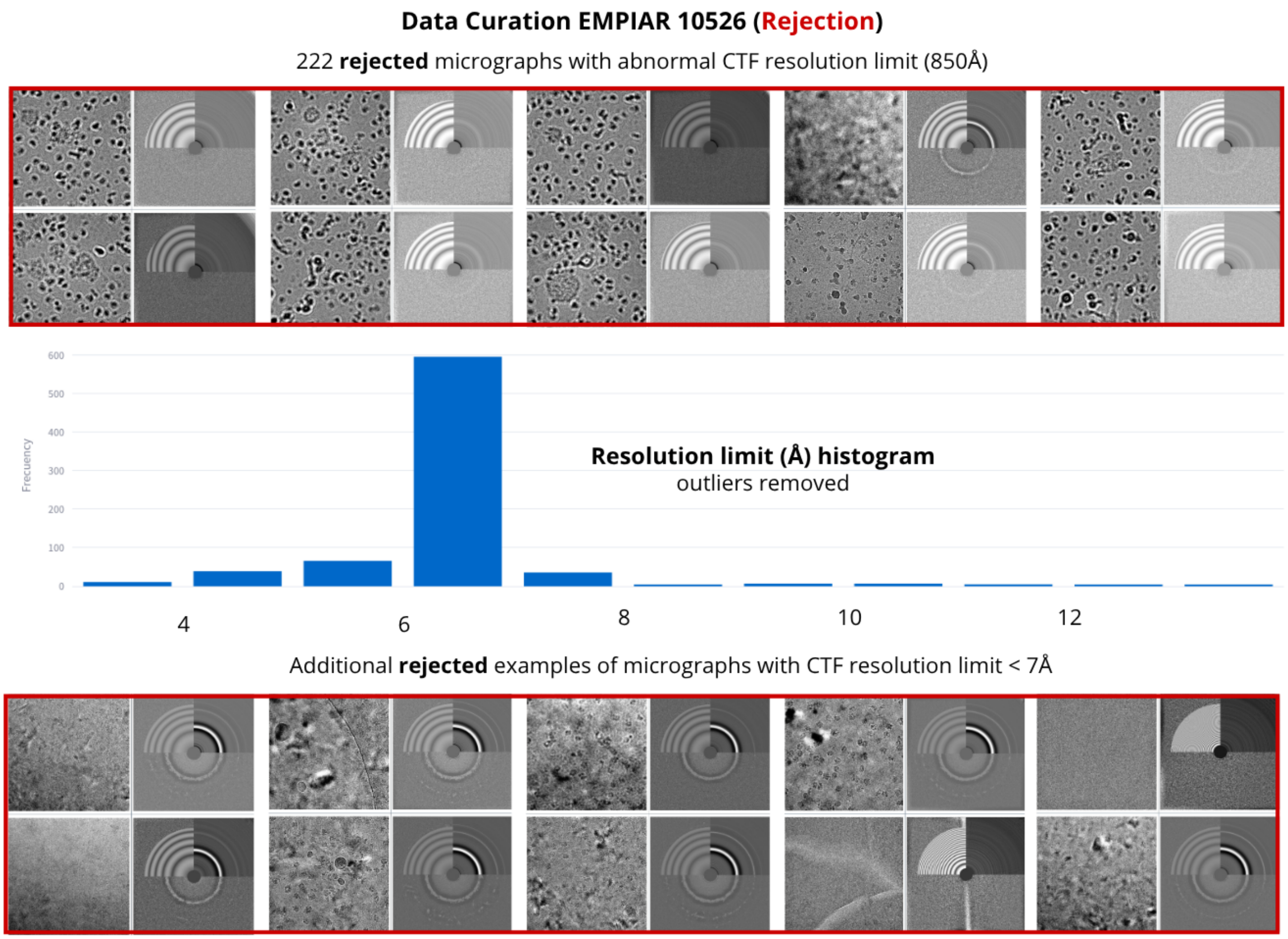

Figure S11: CTF-based data curation analysis for the EMPIAR-10526 dataset. The upper panel shows representative examples of the 222 micrographs rejected due to abnormal CTF resolution estimates, each paired with its corresponding power spectrum on the right. These cases are indica-tive of CTF fitting failures, and visual inspection confirmed the presence of severe crystalline ice contamination, supporting their exclusion from downstream processing. After removing these ex-treme outliers, the resolution-limit histogram shows that most of the remaining micrographs have estimated resolutions between 6 and 7 Å (595 images). Further inspection of micrographs with estimated resolution limits <7 Å (754 images) revealed that many were additionally rejected by other quality-control criteria, including drift, PSD consistency, and MIFFI filtering. Representative examples of these moderately low-quality micrographs are shown in the lower panel, together with their corresponding power spectra.

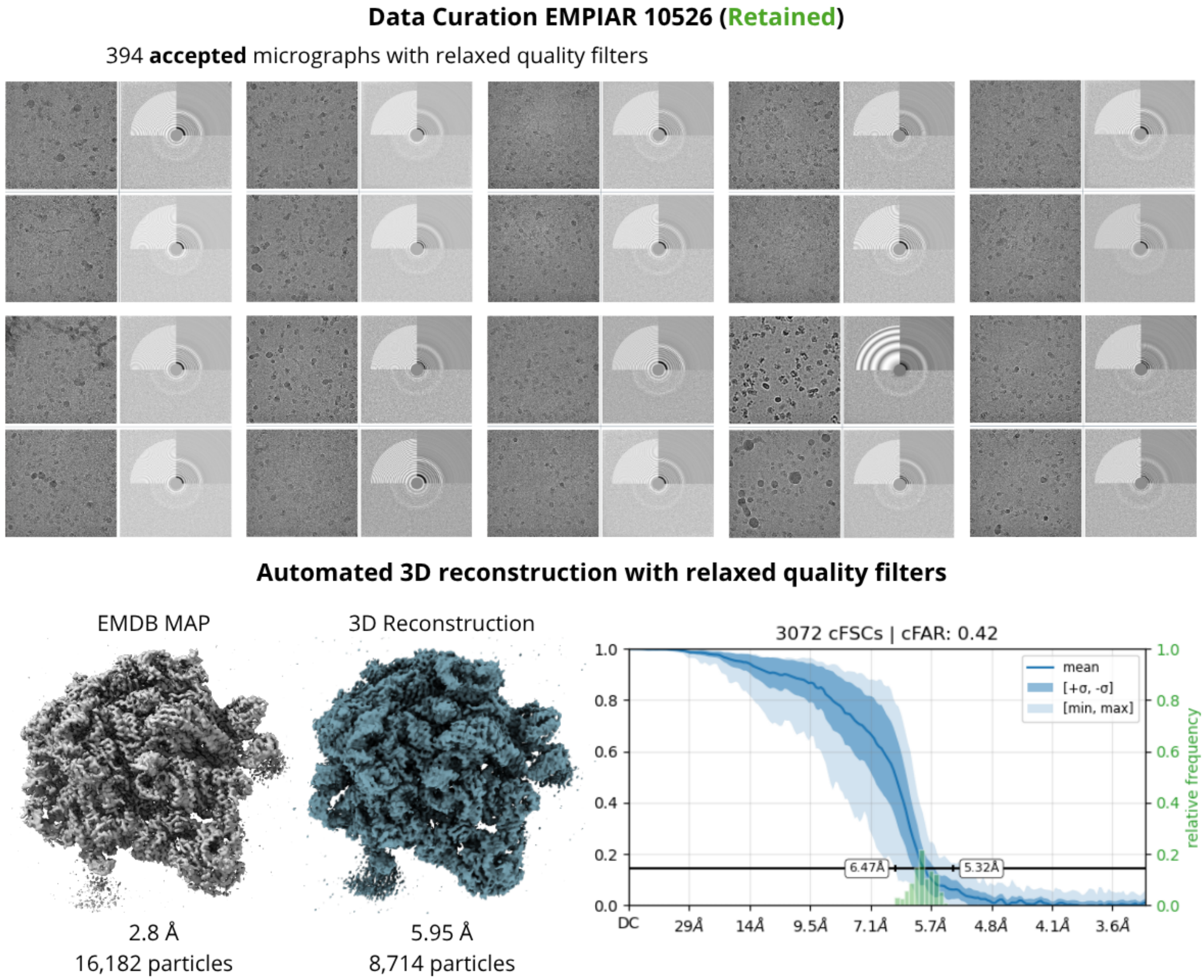

Figure S12: Automated pipeline performance under relaxed filtering conditions for the EMPIAR-10526 dataset. (**Top**) Representative micrographs accepted for downstream processing under re-laxed curation criteria, each paired with its corresponding power spectrum on the right. The relaxed configuration included an increased defocus tolerance (< 55,000 Å), a less stringent CTF resolution threshold (< 7 Å), and PSD analysis adapted for lower-resolution data. (**Bottom**) Comparison between the deposited EMDB map (left) and the 3D reconstruction obtained under relaxed filtering conditions (right). The conical Fourier Shell Correlation (cFSC) curves exhibit narrow standard-deviation bands around the mean, indicating isotropic resolution and uniform directional quality. Despite the lower quality, the reconstructed map preserves the overall structural features of the ref-erence volume, demonstrating the robustness of the pipeline when filtering parameters are adapted to suboptimal datasets.

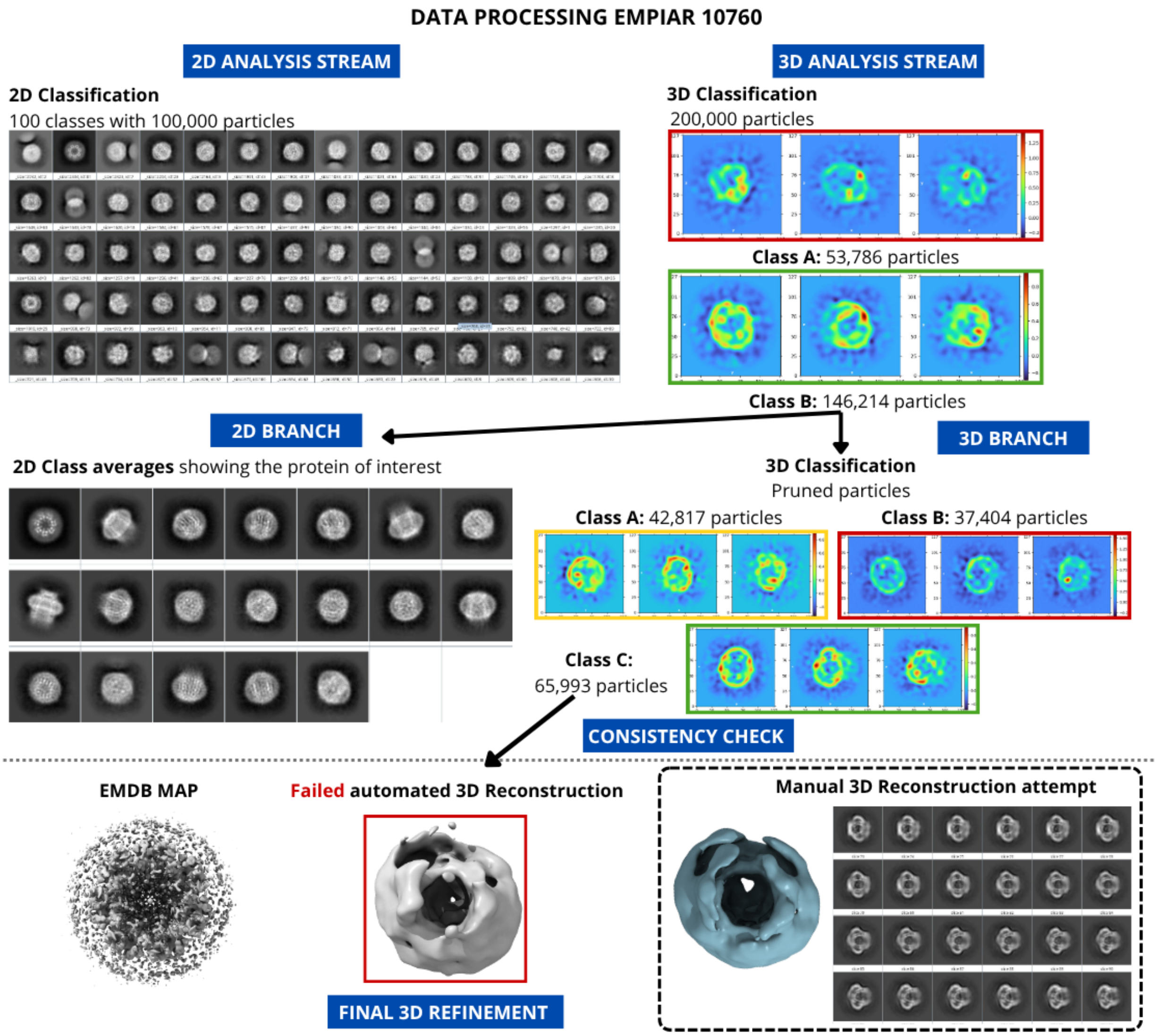

Figure S13: Processing workflow and reconstruction outcome for EMPIAR-10760. The schematic summarizes the automated image processing pipeline from particle picking to the final unsuccessful 3D reconstruction. Each processing stage is indicated by blue boxes, showing the progression of the dataset through the pipeline. Following particle picking, parallel 2D and 3D analysis branches are initiated. The initial 2D classification (100,000 particles) reveals a strong dominance of empty nanodiscs, with only a small fraction of classes displaying protein-like features. In the 3D branch, *ab initio* reconstruction produces a dominant class (146,000 particles) with only a weak resemblance to the expected protein structure. Subsequent rounds of 2D classification during refinement and parallel validation support the selection of this subset, as they yield an increased proportion of protein-like 2D averages. However, in the parallel 3D branch, only one of the newly generated *ab initio* models exhibits a faint protein-like morphology. This volume is selected by the Consis-tency Check step. Despite identifying a subset of particles with weak protein signal, non-uniform refinement fails to converge to an interpretable 3D reconstruction. For comparison, a manual pro-cessing result (dashed lines), following the original study, is also shown. Starting from 200,000 particles, two rounds of 2D classification with manual class selection were performed, followed by two rounds of 3D classification and non-uniform refinement. This approach yielded a reconstruction with slightly improved structural features of the target protein embedded in nanodiscs. However, the final resolution remains limited (7.7 Å), highlighting the intrinsic difficulty of this dataset.
